# The extremely halotolerant black yeast *Hortaea werneckii* - a model for intraspecific hybridization in clonal fungi

**DOI:** 10.1186/s43008-019-0007-5

**Published:** 2019-07-08

**Authors:** Polona Zalar, Jerneja Zupančič, Cene Gostinčar, Janja Zajc, G. Sybren de Hoog, Filomena De Leo, Armando Azua-Bustos, Nina Gunde-Cimerman

**Affiliations:** 1 Department of Biology, Biotechnical Faculty, University of Ljubljana, Večna pot 111, SI-1000 Ljubljana, Slovenia; 2 Lars Bolund Institute of Regenerative Medicine, BGI-Qingdao, Qingdao, 266555 China; 3 Department of Biotechnology and Systems Biology, National Institute of Biology, Večna pot 111, 1000 Ljubljana, Slovenia; 4 Westerdijk Fungal Biodiversity Institute, P.O. Box 85167, Utrecht, 3508 AD The Netherlands; 5 Centre of Expertise in Mycology of RadboudUMC, Canisius Wilhelmina Hospital, Nijmegen, The Netherlands; 6 Department of Chemical, Biological, Pharmaceutical and Environmental Sciences (ChiBioFarAm), University of Messina, Viale F. Stagno d’Alcontres, 31 98166 Messina, Italy; 7 Centro de Astrobiología (CSIC-INTA), Madrid, Spain; 8 Instituto de Ciencias Biomédicas, Facultad de Ciencias de la Salud, Universidad Autónoma de Chile, 8910060 Santiago, Chile

**Keywords:** Morphology, Clustering, NaCl tolerant enyzmes, ITS, D1/D2 rDNA, *MCM7*, *BTB*, Genomes

## Abstract

The polymorphic black yeast *Hortaea werneckii* (*Capnodiales*, *Ascomycota*) is extremely halotolerant (growth from 0 to 30% [w/v] NaCl) and has been extensively studied as a model for halotolerance in Eukaryotes for over two decades. Its most frequent sources are hypersaline environments and adjacent sea-water habitats in temperate, subtropical and tropical climates. Although typically saprobic, *H. werneckii* can also act as a commensal coloniser on human skin, causing *tinea nigra* on hands and soles. Here, we report that addition of NaCl to culture media expands the growth range of *H. werneckii* to 37 °C, which explains its colonisation of human skin, with its increased salinity. The morphological and physiological plasticity/ versatility of *H. werneckii* indicate that a species complex might be involved. This was investigated in this polyphasic taxonomic analysis based on the global diversity of *H. werneckii* strains collected from hypersaline environments, and from humans and animals. Analysis of D1/D2domains of 28S and internal transcribed spacer rDNA revealed 10 and 17 genotypes, respectively, that were not always compliant. The genotypes have global distributions. Human and environmental strains with the same genotypes are intermingled. Due to the limited number of phylogenetically informative characters in the ribosomal DNA dataset, the partial genes encoding for β-tubulin (*BTB*) and mini-chromosome maintenance protein (*MCM7*) were also sequenced. The use of these genes was hampered by ambiguous sequences obtained by Sanger sequencing, as a consequence of the diploid and highly heterozygous genome of many *H. werneckii* strains. Analysis of the *BTB* and *MCM7* genes showed that in some cases two copies of the gene from the same genome are positioned in distant phylogenetic clusters of the intraspecific gene tree. Analysis of whole-genome sequences of selected *H. werneckii* strains generally confirmed the phylogenetic distances estimated on the basis of ribosomal genes, but also showed substantial reticulation within the phylogenetic history of the strains. This is in line with the hypothesis that the diploid genomes of *H. werneckii* were formed by hybridizations, which have sometimes occurred between relatively divergent strains.

**Electronic supplementary material:**

The online version of this article (10.1186/s43008-019-0007-5) contains supplementary material, which is available to authorized users.

## INTRODUCTION

The ascomycetous black yeast *Hortaea werneckii* (*Capnodiales*, *Teratosphaeriaceae*) is the most extremely halotolerant fungus known, and as such, it has become an important model organism for the study of halotolerance in Eukarya (Plemenitaš et al. 2008, 2014, Gunde-Cimerman et al. 2018). Its primary ecological niche are natural hypersaline environments worldwide. It has been isolated from thalasso-haline brines in salterns on three continents, with numerous locations ranging from temperate (Mediterranean) (Gunde-Cimerman et al. 2000, Cabañes et al. 2012, Gunde-Cimerman & Zalar 2014, Elsayed et al. 2016) to subtropical and tropical (Mexico, Puerto Rico) (Díaz-Muñoz & Montalvo-Rodríguez 2005, Cantrell et al. 2006) climate zones, although never in subpolar and polar regions. *Hortaea werneckii* is more frequently found in eutrophic thalasso-haline waters at moderate temperatures, while it is not known to occur in oligotrophic or athalasso-haline hypersaline waters, and only rarely in hypersaline waters with elevated temperatures (Plemenitaš & Gunde-Cimerman 2005). Occasionally, *H. werneckii* has been found in seawater (Iwatsu & Udagawa 1988), on rocks adjacent to seawater (Ruibal et al. 2009), and inhabiting sea sponges (Brauers et al. 2001), corals (Amend et al. 2012, Xu et al. 2014), marine fish (Todaro et al. 1983), salted freshwater fish (Mok et al. 1981), beach soil (de Hoog & Guého 1998), saltern microbial mats (Cantrell et al. 2006), and salt marsh plants (Formoso et al. 2015), and as an endophyte in mangrove plants (Chen et al. 2012). Recently, it was discovered in an indoor dust sample collected in Hawaii (Humphries et al. 2017). In agreement with the extremotolerant nature, it was isolated from 2500 m in depth in the Mediterranean Sea (De Leo et al. 2018), from sediments 5000 m below sea level in the Central Indian Basin (Singh et al. 2012), and in sediments 4000 m below sea level in the East India Ocean (Zhang et al. 2014). Using internal transcribed spacer (ITS) clone libraries, *H. werneckii* operational taxonomic units were detected in the South China Sea in methane-hydrate-bearing deep-sea sediments (Lai et al. 2007). In spite of the wide distribution in marine environments, brines in solar salterns are considered the primary ecological niche, where at salinities above 20% (w/v) NaCl, *H. werneckii* represents from 70 to 80% of all fungal isolates (Gunde-Cimerman et al. 2000; Butinar et al. 2005), with densities of up to 1400 CFU/L.

*Hortaea werneckii* is the only fungus that can grow across almost the whole range of NaCl concentrations, from 0 to 30% NaCl, with a broad optimum between 6 and 14% NaCl (Butinar et al. 2005). As well as NaCl, *H. werneckii* can also tolerate up to 2 M concentrations of the chaotropic salt MgCl_2_, and up to 1.7 M CaCl_2_, and it has been isolated from bitterns, i.e. MgCl_2_-rich leftover brines after harvesting of NaCl (Zajc et al. 2014). The species also grows on osmotic media with 10% NaCl with 12% glucose (e.g. malt extract, yeast extract, 10% NaCl, 12% glucose agar) and with 50% glucose (e.g., malt extract, yeast extract, 50% glucose agar) (Gunde-Cimerman et al. 2000, Humphries et al. 2017).

Until 2000, *H. werneckii* was primarily known as the aetiological agent of a skin disorder known as *tinea nigra*, a superficial infection of the human hands and feet that can occur in warmer climates (de Hoog et al. 2000, Perez et al. 2005, Bonifaz et al. 2008). Infections have been associated with superficially abraded skin exposed to hypersaline tidal waters (de Hoog & Gerrits van der Ende 1992) in combination with hyperhydrosis (Bonifaz et al. 2008, Paneque Rodríguez et al. 2015). The species has been associated with animal bites (Rossetto et al. 2014). The pathogenic potential of *H. werneckii* has, however, remained unclear, as it was generally considered that the species could not grow at 37 °C (de Hoog et al. 2000, Ng et al. 2005).

Under extremely saline conditions, *H. werneckii* has an extremophilic ecotype, which is characterised by enhanced melanization, changes in cell size and meristematic growth, and changes in colony appearance (Kogej et al. 2007). At the molecular level, the responses of *H. werneckii* to high concentrations of toxic sodium ions have involved changes in the expression of genes associated with the synthesis of compatible solutes, regulation of intracellular alkali-metal cation concentrations, modifications of cell membrane lipid composition, and changes in cell-wall ultrastructure and morphology (Plemenitaš et al. 2014). Due to these adaptations, *H. werneckii* has an outstanding ability to overcome the turgor loss and sodium toxicity that are typical of hypersaline environments (Gostinčar et al. 2011).

Genome sequencing of the *H. werneckii* strain EXF-2000 (Lenassi et al. 2013, Sinha et al. 2017) has revealed that with almost 50 Mb, the *H. werneckii* genome is larger than the genomes of other sequenced representatives of *Capnodiales*. The *H. werneckii* genome contained close to 16,000 genes, of which 90% were present in two copies, with an average of 5% amino-acid sequence divergence between the predicted proteins. Genomic data fit well with the molecular studies on individual genes performed earlier, as all of the genes studied to this point were duplicated and coded for two functionally identical proteins (Vaupotič & Plemenitaš 2007, Vaupotič et al. 2008).

Recent analyses of the whole genome sequences of 11 additional strains revealed that two were haploid and the remaining nine were diploid. Differences between the subgenomes of individual strains and their detailed phylogenetic analyses have shown that the diploid genomes were most likely produced by hybridization between relatively divergent strains (Gostinčar et al. 2018). At the same time, apart from identification of a heterothallic mating locus, the comparative genomic analyses have failed to find any traits that can be linked to sexual reproduction.

In summary, the global presence of *H. werneckii* in hypersaline environments, its role as the most important model for halotolerance in *Eukarya*, its extensive physiological plasticity and adaptability, the availability of numerous strains collected from various osmotic environments, and its particular combination of hybridization with an exclusively or primarily clonal reproduction mode (as revealed by comparative genomics) make *H. werneckii* an attractive model organism.

The present study compared 98 strains, of which a selection was subjected to classical taxonomic analyses (macro-, microscopy, assimilation tests), and was tested for halotolerance and thermotolerance and the production of selected pathogenicity-related enzymes (proteases, esterases), both with and without the addition of salt. Taxonomic analyses have been supported by comparative analyses of the most frequently used barcodes, ITS and D1/D2 28S rDNA. Indeed, apart from the ribosomal regions, almost all of the other genes were present in two different copies per strain, which prevented classical taxonomic phylogenetic approaches based on analyses of household genes. Therefore, in addition to ambiguous Sanger sequences and adjusted algorithms to work with such sequences, sequences derived from whole-genome sequencing of 11 strains were also considered. The taxonomic study presented was aimed at definition of the variability in *H. werneckii*, the description of potentially new species, and the definition of genotypes that might correlate to particular ecological habitats.

## METHODS

This study presents a taxonomic analysis of 98 strains (Table 1) that were mostly isolated from brine or bitterns of three Mediterranean salterns (47 strains), adjacent sea and deep sea environments (10 strains), and air above hypersaline ponds (1). Five strains were also included from patients living in the Mediterranean area (i.e., Italy, Portugal, France), as well as strains from animals (7) and plants (3). One strain was food-borne (it formed a black biofilm on cheese brine), one originated from the air in Japan, and one was of unknown source. Twenty-two strains originated from a coastal cave located in the Coastal Range of the Atacama Desert (Chile). These strains were isolated from spider webs attached to the cave wall, from a newly described species of microalga (*Dunaliella atacamensis*; Azúa-Bustos et al. 2010), and directly from the cave walls and from sand underneath the spider webs.

**Table 1:** *Hortaea werneckii* strains treated in this study

Strain no.		ORIGIN (sample, collection date, location)		GenBank acc. No.	
EXF-	Other collections		Genotype (28S/ITS)	D1/D2 28S rDNA	ITS rDNA
Hypersaline brine and bittern in salterns					
9		brine, 11/1998, Salinas de la Trinitat (Ebre Delta), Spain	D/8	MH327587	MH327684
64		brine, 7/1998, Salinas de la Trinitat (Ebre Delta), Spain	B/7	MH327569	MH327666
96		brine, 11/1998, Salinas de la Trinitat, Spain	D/8	MH327585	MH327682
120		brine, pond 3, 11/1998, Salinas Santa Pola, Spain	B/7	MH327575	MH327672
132		brine, pond 5, 11/1998, Salinas Santa Pola, Spain	B/7	MH327576	MH327673
152		brine, pond 1, 9/1996, Sečovlje salterns, Slovenia	B/7	MH327577	MH327674
153		brine, pond 1, 5/1996, Sečovlje, Slovenia	B/7	MH327597	MH327694
154		brine, pond 1, 9/1996, Sečovlje, Slovenia	B/7	MH327581	MH327678
225		brine, pond 1, 8/1996, Sečovlje, Slovenia	B/7	MH327568	MH327665
241		brine, pond 1, 6/1996, Sečovlje, Slovenia	A/1	MH327539	MH327636
247		brine, pond 1, 9/1996, Sečovlje, Slovenia	A/1	MH327541	MH327638
269		brine, 11/1998, Santa Pola, Spain	B/7	MH327579	MH327676
489		brine, pond 3, 11/1998, Salinas de la Trinitat, Spain	C/9	MH327592	MH327689
537		brine, pond 1, 8/1996, Sečovlje, Slovenia	B/7	MH327571	MH327668
554		brine, pond 1, 8/1996, Sečovlje, Slovenia	A/1	MH327532	MH327629
561		brine, 3/2000, Salterns at the Skeleton coast (Atlantic), Namibia	J/13	MH327618	MH327626
562		brine, 3/2000, Salterns at the Skeleton coast (Atlantic), Namibia	E/4	MH327563	MH327660
631		brine, 11/1998, Santa Pola, Spain	D/8	MH327586	MH327683
647		brine, 11/1998, Santa Pola, Spain	D/7	MH327564	MH327661
2516		brine, 12/2004, Candelaria, Puerto Rico	I/11	MH327546	MH327643
2685	CBS 100456	brine, 1998, Sečovlje, Slovenia	B/7	MH327590	MH327687
2782		brine, pond 4, 8/1996, Sečovlje, Slovenia	B/7	MH327598	MH327695
2783		brine, pond 2, 8/1996, Sečovlje, Slovenia	D/8	MH327596	MH327693
2785		brine, pond 1, 5/1996, Sečovlje, Slovenia	C/9	MH327593	MH327690
2787		immersed wood in brine, pond 1, 1/1997, Sečovlje, Slovenia	B/7	MH327580	MH327677
2788		brine, pond 2, 7/1996, Sečovlje, Slovenia	D/8	MH327584	MH327681
3506	PR 1105-73	brine, Candelaria, Puerto Rico	E/5	MH327550	MH327647
3846	PR08-Candel.12	brine, 3/2008, Candelaria, Puerto Rico	A/16	MH327545	MH327642
4642		brine, pond 1, 9/1996, Sečovlje, Slovenia	B/7	MH327572	MH327669
4662		brine, pond 1, 9/1996, Sečovlje, Slovenia	B/7	MH327573	MH327670
4667		brine, pond 1, 7/1996, Sečovlje, Slovenia	B/7	MH327574	MH327671
4717		brine, pond 1, 8/1996, Sečovlje, Slovenia	B/10	MH327591	MH327688
7620		brine, reservoir, 12/2009, Sečovlje, Slovenia	B/7	MH327578	MH327675
7637		brine, reservoir, 12/2009, Sečovlje, Slovenia	B/7	MH327566	MH327663
7638		brine, reservoir 12/2009, Sečovlje, Slovenia	B/7	MH327565	MH327662
10304		brine, 11/2015, Sečovlje salterns, Slovenia	B/7	MH327600	MH327698
10813		bittern, 11/2016, Sečovlje salterns, Slovenia	B/7	MH327601	MH327699
10820		bittern, 11/2016, Sečovlje salterns, Slovenia	B/7	MH327582	MH327679
10828		brine, 11/2016, Sečovlje salterns, Slovenia	A/1	MH327602	MH327700
10830		brine, 11/2016, Sečovlje salterns, Slovenia	D/8	MH327588	MH327685
10831		brine, 11/2016, Sečovlje salterns, Slovenia	B/8	MH327603	MH327701
10834		brine, 11/2016, Sečovlje salterns, Slovenia	B/7	MH327604	MH327702
10842		brine, 11/2016, Sečovlje salterns, Slovenia	C/9	MH327595	MH327692
10843		brine, 11/2016, Sečovlje salterns, Slovenia	B/9	MH327605	MH327703
10957		bittern, 11/2016, Sečovlje salterns, Slovenia	B/7	MH327607	MH327705
10974		brine, 11/2016, Sečovlje salterns, Slovenia	C/9	MH327608	MH327706
10975		brine, 11/2016, Sečovlje salterns, Slovenia	C/9	MH327609	MH327707
Sea water and related habitats					
166	CBS 100496	sea water-sprayed marble, Delos, Greece	A/1	MH327538	MH327635
2684		sea water, 1998, Slovenia	C/9	MH327594	MH327691
2686	CBS 373.92	beach sand, 1992, La Palma, Spain	H/1	MH327530	MH327627
10508	AT 25f	Mediterranean sea, depth 25 m, 12/2013, “Atalante” station	E/4	MH327555	MH327652
10509	KM3 200r	Mediterranean sea, depth 200 m, 12/2013, “KM3” station	E/4	MH327556	MH327653
10510	M 94	Mediterranean sea, depth 94 m, 12/2013, “Medee” station	E/4	MH327557	MH327654
10511	V 25 a	Mediterranean sea, depth 25 m, 12/2013, “Vector” station	E/4	MH327558	MH327655
10512	V 25c	Mediterranean sea, depth 25 m, 12/2013, “Vector” station	E/4	MH327559	MH327656
10513	V 2500b	Mediterranean sea, depth 2 500 m, 12/2013, “Vector” station	E/4	MH327560	MH327657
10853	no. 445345	beach sand, Portugal	B/7	MH327606	MH327704
Animal related strains					
156	CBS 116.90	fish kantar (*Spondyliosoma cantharus*), eye infection, unknown	B/7	MH327570	MH327667
157	CBS 115.90	frog, kidney, Brazil	E/4	MH327554	MH327651
2683	CBS 117.90	fish *Osteoglossum bicirrhosum*, Brazil	A/1	MH327542	MH327639
4625	CBS 100455	red coral (*Corallium rubrum*), 8/1996, Ugljan, Croatia	B/7	MH327567	MH327664
12619	MCCC 3A00558	corals, Pacific Ocean, China	A/5	MH327619	MH327714
12620	MCCC 3A00680	corals, Pacific Ocean, China	E/15	MH327620	MH327716
12684	MCCC 3A00555	corals, Pacific Ocean, China	A/5	MH327621	MH327715
Plant related strains					
161	CBS 706.76	unknown tree leaf, Senegal	D/8	MH327583	MH327680
2688	CBS 255.96	*Casuarina equisetifolia*, Canarian Islands, Spain	B/12	MH327589	MH327686
2690	CBS 707.76	sooty mould, Sri Lanca	F/4	MH327552	MH327649
Human related strains					
151	CBS 107.67 ^T^	man, tinea nigra, Portugal	G/1	MH327544	MH327641
155	CBS 359.66	man, unknown	A/1	MH327543	MH327640
171	CBS 111.31	man, keratomycosys, Brazil	E/4	MH327551	MH327648
177	CBS 705.76	man, tinea nigra, France	A/1	MH327540	MH327637
2682	CBS 126.35	man, trichomycosys nigra, 9/1935, Italy	F/6	MH327561	MH327658
Strains from arid environments					
6651		spiderweb with (w) algae, 1/2010, Huanillos cave, Atacama, Chile	A/1	MH327533	MH327630
6652		spiderweb w algae, 1/2010, Huanillos cave, Atacama, Chile	A/1	MH327534	MH327631
6653		spiderweb w algae, 1/2010, Huanillos cave, Atacama, Chile	A/1	MH327535	MH327632
6654		spiderweb without (wo) algae, 1/2010, Huanillos cave, Atacama, Chile	A/3	MH327549	MH327646
6655		spiderweb wo algae, 1/2010, Huanillos cave, Atacama, Chile	A/1	MH327536	MH327633
6656		rock, 1/2010, Huanillos cave, Atacama, Chile	A/2	MH327547	MH327644
6658		spiderweb wo algae, 1/2010, Huanillos cave, Atacama, Chile	A/1	/	MH327722
6663		spiderweb w algae, 1/2010, Huanillos cave, Atacama, Chile	A/2	MH327622	MH327718
6664		spiderweb w algae, 1/2010, Huanillos cave, Atacama, Chile	A/1	MH327531	MH327628
6665		spiderweb w algae, 1/2010, Huanillos cave, Atacama, Chile	A/3	MH327548	MH327645
6666		spiderweb w algae, 1/2010, Huanillos cave, Atacama, Chile	A/2	MH327623	MH327719
6667		spiderweb w algae, 1/2010, Huanillos cave, Atacama, Chile	A/17	MH327625	MH327723
6668		spiderweb w algae, 1/2010, Huanillos cave, Atacama, Chile	A/2	MH327624	MH327720
6669		spiderweb w algae, 1/2010, Huanillos cave, Atacama, Chile	A/1	MH327537	MH327634
11528		sand, 2/2017, Huanillos cave, Atacama, Chile	A/1	MH327610	MH327721
11531		sand, 2/2017, Huanillos cave, Atacama, Chile	A/2	MH327611	MH327708
11537		sand, 2/2017, Huanillos cave, Atacama, Chile	A/14	MH327612	MH327709
11538		sand, 2/2017, Huanillos cave, Atacama, Chile	A/2	MH327613	MH327710
11539		sand, 2/2017, Huanillos cave, Atacama, Chile	A/2	MH327614	MH327711
11540		sand, 2/2017, Huanillos cave, Atacama, Chile	A/2	MH327615	MH327712
11547		sand, 2/2017, Huanillos cave, Atacama, Chile	A/2	MH327616	/
11548		sand, 2/2017, Huanillos cave, Atacama, Chile	A/1	MH327617	MH327713
2687	CBS 410.51	air, 2/1951, Japan	F/4	MH327553	MH327650
4493		air, 1993, abandoned salterns Sečovlje, Slovenia	nd/10	/	MH327696
Other					
4661	CBS 122.32	unknown	F/6	MH327562	MH327659
8422		biofilm from salted water of a cheese factory, Slovenia	B/7	MH327599	MH327697

### Isolation of strains and sampling sites

The *Hortaea werneckii* strains studied are listed in Table 1, and were collected over a 20-year period. They are maintained in the Microbial Culture Collection Ex of the Infrastructural Centre Mycosmo, MRIC UL, Slovenia (http://www.ex-genebank.com), in the Department of Biology, Biotechnical Faculty, University of Ljubljana (Slovenia). Most of these strains originated from brine and bitterns of the Sečovlje seasonal solar salterns on the northern Adriatic coast (Zalar et al. 1999, Gunde-Cimerman et al. 2000, Butinar et al. 2005), at the border between Slovenia and Croatia (sub-Mediterranean climate). Brines from salterns in Spain (i.e. Santa Pola and Ebro River Delta) were sampled in 1999 and 2000, and in Namibia (i.e. Skeleton coast) in 2003. Isolations were performed as described in Gunde-Cimerman et al. (2000), by filtration of water and placing the filters on hypersaline culture media. Strains from bitterns were isolated with additional enrichment in half-strength liquid media: malt extract (pH 7, 3.5), yeast nitrogen base (YNB), and corn steep liquid supplemented with 50 mg/L chloramphenicol. Enrichments were carried out in 500 mL Erlenmeyer flasks with 50 mL medium and 50 mL original bittern. After 7 d incubation on a rotary shaker at 25 °C at 180 rpm, 100 μL of the broth was cultured on DG18, malt extract agar (MEA) + 3 M NaCl and MEA + 1.5 M MgCl_2_.

The strains from Puerto Rico salterns originate from the study of Cantrell et al. (2006). Seawater and deep sea strains were collected in 2013 during the DEEP-PRESSURE Cruise on board the R/V Urania (Smedile et al. 2015), from water columns 25–2500 m in depth, sampled at four stations in the Mediterranean Sea: Station Vector in the Tyrrhenian Sea (39°32′00.6″N 13°22′28.5″E, Station KM3 in the Ionian Sea (36°30′61.3″N 15°40′59.9″E), and above the anoxic hypersaline lakes L’Atalante (35°18′92″N 21°23′92″E) and Medee (34°24′00″N 22°26′99″E), in the central and south Mediterranean Sea, respectively (de Leo et al. 2018). Strains from corals in the Pacific Ocean (China) were isolated by Luo et al. (2017). A large number of strains were collected in 2009 and 2017 by Azúa-Bustos from soil, sand and spider webs collected inside Huanillos cave, which is located under an ancient guano deposit that is rich in ammonium nitrate, urate and phosphates. This cave is in the Coastal Range hills of the Atacama Desert, south of the city of Iquique, Chile (Azúa-Bustos et al. 2010), as also described by Martinelli et al. (2017).

All of the strains were single colony isolates after plate streaking. All of the human-, animal-, and plant-related strains were obtained from the Westerdijk Institute for Fungal Biodiversity culture collection (CBS; http://www.westerdijkinstitute.nl/Collections/).

### DNA extraction and sequencing

DNA extraction was performed according to van den Ende & de Hoog (1999). About 50 ng template DNA was used in the PCRs. The complete ITS and the D1/D2 domain of 28S rDNA were amplified with the primers ITS4 and ITS5 (White et al. 1990) or NL1 and NL4 (O’Donnell 2000). A fragment of the β-tubulin gene (*BTB*) was amplified with primers BT2a and BT2b (Glass & Donaldson 1995), and a fragment of DNA replication licensing factor required for DNA replication initiation and cell proliferation (*MCM7*) was amplified with primers Mcm7-709for and Mcm7-1348rev (Schmitt et al. 2009). The same primers were also used in the sequencing reactions. Sequences were generated through Sanger sequencing (16 ABI 3730xl) using a commercial provider (Microsynth, Wien, Austria). The sequence chromatograms were manually checked for quality using the FinchTV software (Geospiza, Los Altos, CA) and assembled and edited using SeqMan 3.61 (DNAStar, Madison, QI). PCR products that resulted in Sanger-derived *BTB* sequences that showed ambiguous signals were first purified from 1% agarose gels with GeneJET Gel extraction kits (ThermoFisher Scientific, Waltham, MA), and then cloned into the pJET vector and transformed into XL1Blue *Escerichia coli* using TransformAid bacterial transformation kits (ThermoFisher Scientific). Transformants were selected after overnight growth on Luria-Bertani agar medium supplemented with ampicillin. Plasmids were extracted from 20 transformant colonies using GeneJET plasmid miniprep kits (ThermoFisher Scientific) and the cloned *BTB* fragments were sequenced as described above. The DNA sequence data have been deposited with GenBank (Table 1).

### ITS and 28S rDNA genotypes and genome variants

ClustalW aligned ITS and 28S rDNA Sanger sequences were checked for single nucleotide polymorphisms and overall similarities using the unweighted pair group method with arithmetic averages (UPGMA) for cluster analyses. MEGA 5.2 was also used to select the nucleotide substitution model used in the UPGMA runs. Other settings included the use of gaps or missing data as partial deletions with a site coverage cut-off of 95%, with nearest-neighbour-interchange used as a heuristic method. Internal branch support was assessed on the basis of 1000 bootstrapped datasets. Genotypes encountered in the ITS were numbered 1–17, and those for the 28S rDNA A–J. Genotypes that were based on both loci were characterised by using first the letter code for the 28 rDNA followed by a slash and then the number code of the ITS. The ITS (28S rDNA) alignment started with ATCATTA (ACGGCGA).

Tandem repeated ITS and 28S rDNA fragments from whole genome sequences were aligned with the ITS and 28S rDNA reference sequences from strain EXF-2000 (= EXF-225) with BWA-MEM (Li & Durbin 2009). Variant calling was performed with Samtools 1.5 (Li et al. 2009) in the diploid mode, after the sequences were sorted and deduplicated.

### Phylogenetic analyses based on protein-encoding genes and whole genomes

The two protein-encoding genes were separately analysed phylogenetically after their sequences were aligned with MAFFT, in automatic mode (Katoh & Toh 2008). Sanger sequences of *BTB* not showing ambiguous bases, which included those with clean chromatograms obtained after cloning, were aligned with *BTB* fragments obtained from whole genomes. Alignments of the *MCM7* sequences were based on sequences without ambiguous signals and also with those showing ambiguous signals, from which the estimated haplotypes were generated using PHASE 2.1 (Stephens et al. 2001), using 10 haplotype pairs from whole genome sequences as known phases (Gostinčar et al. 2018). The resulting haplotype pairs that showed identical tree positions in preliminary phylogenetic analyses were collapsed into one sequence. The *MCM7* alignments also included sequences derived from sequenced genomes. Best suiting substitution models were determined using jModelTest 2.1.10 (Darriba et al. 2012) and phylogenetic trees were generated using PhyML 3.1 (Guindon et al. 2010). The alpha parameter of the gamma distribution of substitution rate categories and the proportion of invariable sites were estimated using PhyML. Branch supports were estimated using aLRT, as Chi2-based supports.

A whole genome phylogenetic network was calculated as described by Gostinčar et al. (2018). In short, the sequencing reads (Genbank BioProject accession code PRJNA42832) were mapped to the genome of strain EXF-562 using BWA-MEM and sorted and deduplicated (Li & Durbin 2009). Variant calling was performed using Genome Analysis Toolkit 3.8 (Alkan et al. 2011) according to ‘GATK Best Practices’ with the ‘hard filtering’ option. After discarding all of the variants with depth of coverage outside the range of 700–900, the resulting single nucleotide polymorphisms were used to calculate the dissimilarity distance matrix with the R package Poppr (Kamvar et al. 2015; R Development Core Team 2017). The Neighbour-Net algorithm as implemented in the R package Phangorn (Schliep et al. 2017) was used for construction of the phylogenetic network, with *Hortaea thailandica* as an outgroup.

All of the alignments have been submitted to TreeBASE, and are accessible at the following address: http://purl.org/phylo/treebase/phylows/study/TB2:S24174

### Halotolerance and thermotolerance tested on solid and in liquid media

The strains characterised in this study were line-streak cultivated on MEA medium supplemented with NaCl (0, 5, 10, 15, 20, 25, and 30%; w/v), and incubated at 25 °C for up to 9 weeks. The colonies were then measured and the colony structures characterised, as yeast (Y), filamentous (F), or yeast-filamentous (Y-F). The temperature tolerance of the strains was tested on MEA medium incubated at 5, 10, 15, 25 and 37 °C for up to 2 weeks. Growth was recorded as good, weak, or no growth.

For testing of the growth at different NaCl concentrations, cells of *H. werneckii* were grown in defined YNB medium (ForMedium, Norfolk, UK): 0.17% (w/v) yeast nitrogen base, 0.5% (w/v) ammonium sulphate, 2.0% (w/v) glucose, in deionised water, with pH adjusted to 7.0, and supplemented with 5, 10, 15, 20, and 25% w/v NaCl, at 24 °C and 37 °C. The tests were performed in 96-well microtiter plates, in a 200 μL volume, with 16 replicates per strain. Each well was inoculated with 10 μL cell suspension (10^7^ cells/mL) obtained from 5-day-old cultures grown on Yeast Extract Peptone Dextrose (YPD) agar (1% yeast extract, 2% peptone, 2% D-glucose, 1.6% agar). The microtiter plates were sealed with parafilm and incubated for up to 2 weeks. Growth was followed daily by measuring the optical density at 590 nm.

### Morphological studies

Strains were streak plated from single colonies onto MEA without NaCl or supplemented with 10% NaCl (MEA + 10% NaCl), onto potato dextrose agar (PDA), and onto oatmeal agar (OA), and incubated at 24 °C for up to 2 weeks. The diameters of the colonies and their colours and structures were recorded after 2 weeks of incubation. The colour codes used to describe the colony colours were from Kornerup and Wanscher (1978). For the microscopy study, slides were prepared from 2-week-old cultures using the media indicated above, from the colony centre and the colony margin, and were mounted in 60% lactic acid. The preparations were observed under light microscopy (BX-51, Olympus, Tokyo) with interference contrast optics. Micrographs were recorded with a digital camera (DP73, Olympus). Conidia (one celled, two celled) and hyphae were measured using the micro-imaging software cellSens (Olympus). These measurements are given as means ±standard deviation from ≥ 50 conidia and ≥ 10 hyphae.

### Use of carbon and nitrogen sources tested with a Biolog system

For phenotypic assessment of five strains of *Hortaea werneckii* (EXF-151, EXF-225, EXF-2788, EXF-6651, and EXF-10513) three different sets of phenotypic microarrays were used (Biolog Inc., Hayward, CA): PM1 MicroPlate and PM2A MicroPlate for carbon metabolism, and PM3B MicroPlate for nitrogen metabolism. The cultures were grown on YPD medium for 5 days at 24 °C.

The cells were then resuspended in sterile water to obtain homogenous suspensions the turbidities of which were adjusted to an absorbance of 0.2 at 590 nm, as described previously (Viti et al. 2015). The inoculation suspensions were prepared by adding 0.25 mL cell suspension in minimal medium (IFY-0, 1.2×) that lacked sources of either carbon (for PM1, PM2A) or nitrogen (for PM3B), and one of the two corresponding phenotypic microarray (12×) additives and tetrazolium dye mix (DyeMixE; Biolog Inc., Hayward, CA). The phenotypic microarray additives were prepared as sterile 12× concentrated solutions: for PM1 and PM2A, the NPS additive (nitrogen/ phosphorus/ sulphur; 60 mM L-glutamic acid monosodium, 60 mM potassium phosphate monobasic anhydrous, pH 6.0, 24 mM sodium sulphate); and for PM3B, the CPS additive (carbon/ phosphorus/ sulphur; 1200 mM D-glucose, 60 mM potassium phosphate monobasic anhydrous, pH 6.0, 24 mM sodium sulphate). The phenotypic microarray plates were wrapped with parafilm and incubated at 24 °C for > 2 weeks, and the absorbance was measured at 590 nm (A_590_) on CytationI3 Imaging reader employing Gen5 Microplate Reader and Imager Software, both from BioTek Instruments (Bad Friedrichshall, Germany). The absorbance readings were taken after 0, 2, 7, and 17 days incubation, and the measurements were compared to the negative control (i.e. inoculated well without carbon and nitrogen source, for PM1–2 and PM3, respectively) and to the measurement of the individual wells before incubation of the phenotypic microarray. The phenotypic microarray tests were interpreted as positive for A_590_ > 0.2, weak for A_590_ 0.1 to 0.2, and negative for A_590_ < 0.1. Additionally, the plates were inspected visually to ensure that the change in absorbance corresponded to the change of colour due to the reduction of the tetrazolium dye.

### Enzyme activity tests

The extracellular enzyme activities were tested on solid 2% agar media (Paterson & Bridge 1994, Strauss et al. 2001, Brizzio et al. 2007) without NaCl and with addition of 10 and 20% NaCl. Conidial suspensions of 7-day-old cultures grown at 24 °C on MEA were used for three-point inoculation of the culture medium. The cultures were incubated in the dark at 25 °C for 2 weeks and 4 weeks. Proteolytic activity was determined on 0.5% skimmed milk agar medium with 0.1% yeast extract (Biolife). A clear zone in the otherwise opaque medium indicated a positive reaction (Brizzio et al. 2007). Esterase activity was determined on Tween 80 agar medium that consisted of 1% Tween 80 (polyoxyethylene-sorbitan-monooleate), 1% peptone (Merck), and 0.01% CaCl_2_ × 2H_2_O. The pH indicator bromocresol blue (12.5 mg/L) was added to the medium before adjusting the pH to 5.4. After incubation, the cleavage of the ester bonds was detected as a precipitate and a purple halo around colonies in the otherwise yellowish-green medium (Brizzio et al. 2007). The basic medium for the testing β-glucosidase was 0.67% YNB (Difco), with the addition of 1% aesculin (Sigma). If not stated otherwise, the pH of all of the media was adjusted to 5.5 before pouring. To determine β-glucosidase activity, filter sterilised 1% ammonium ferric citrate solution was added to the medium, to a final concentration of 0.02%. Enzymatic activity was recognised by dark brown coloration of the medium (Strauss et al. 2001). The activity of gelatinases was determined in 5 mL nutrient gelatine tubes with 12% gelatine (Sigma-Aldrich, Munich, Germany), 0.3% beef extract (Becton Dickenson, Franklin Lakes, NJ), and 0.5% peptone (Merck, Darmstadt, Germany). After the incubation, the tubes were placed at 4 °C for 30 min, to allow liquefaction of the gelatine, which was interpreted as a positive reaction (Hankin & Anagnostakis 1975).

### Statistical analysis

The Biolog data were tested for similarity using the SIMPER test (Euclidean distance index) and the PAST software, version 3.13. The phenotypic data (temperature, ESTs) were subjected to multivariant ordination principal component analysis using the PAST software, version 3.13 (Hammer et al. 2001).

## RESULTS

### Cluster analysis

**Fig. 1:**
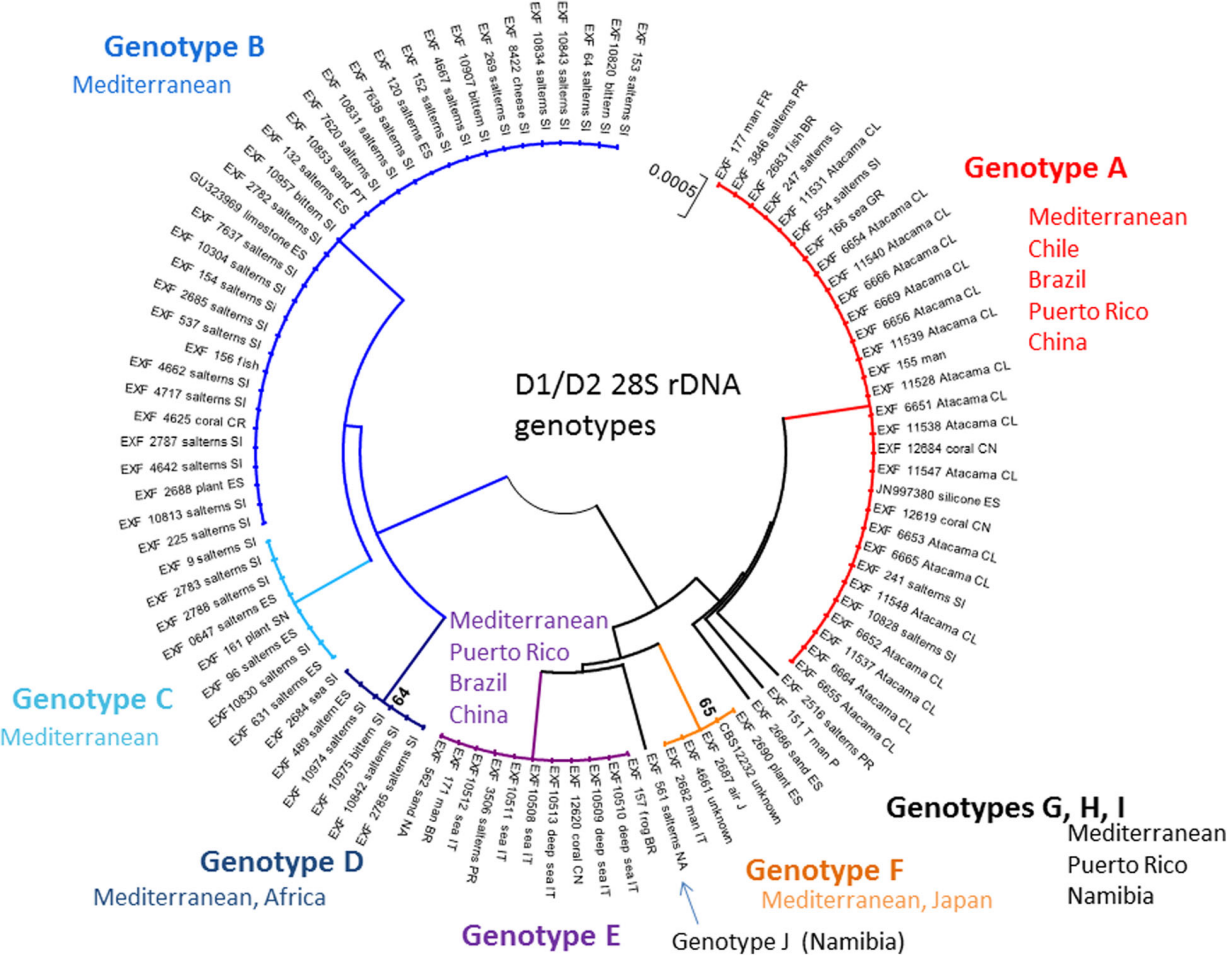
Unweighted pair group method with arithmetic averages analysis of *Hortaea werneckii* D1/D2 28S rDNA sequences defines 10 genotypes

**Fig. 2:**
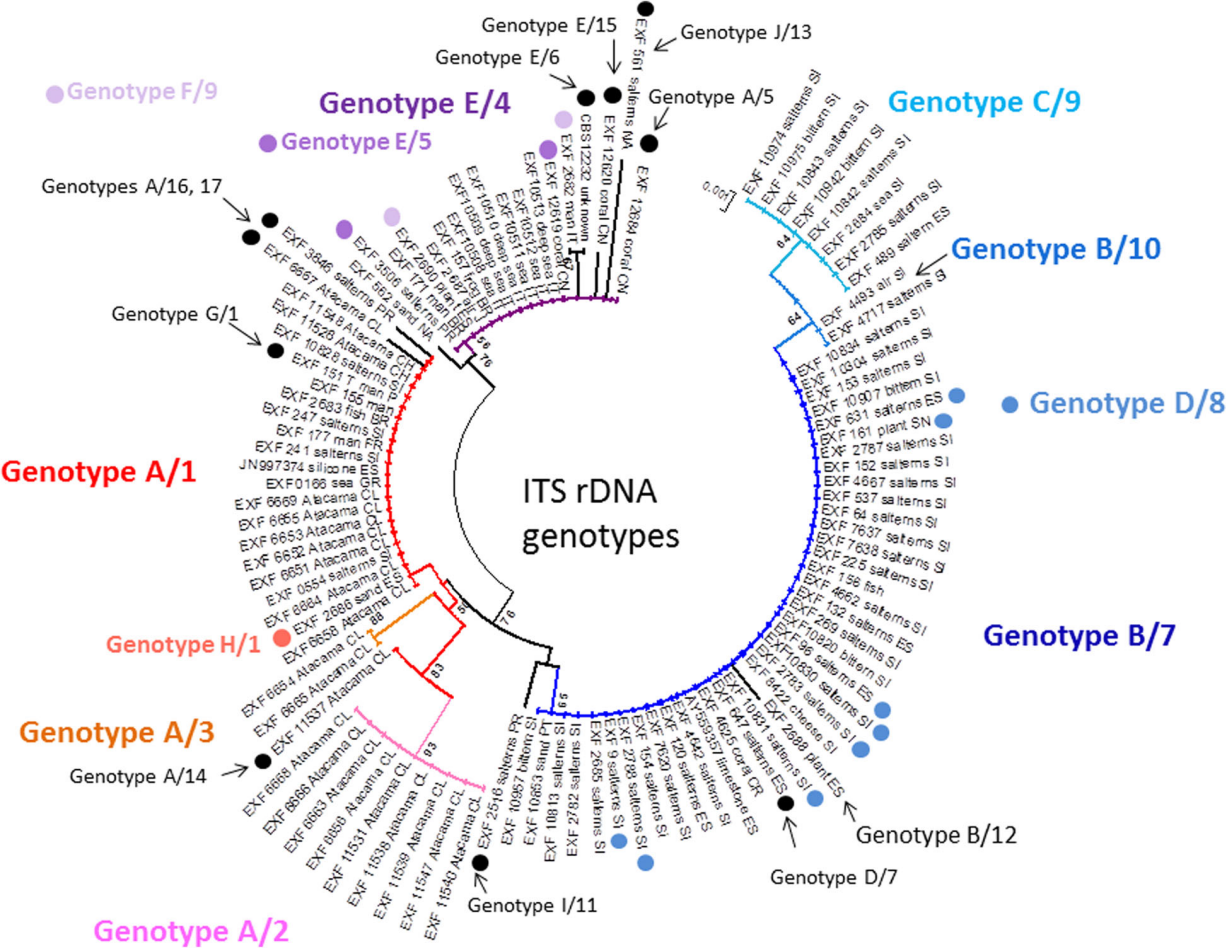
Unweighted pair group method with arithmetic averages analysis of *Hortaea werneckii* ITS rDNA genotypes defines 17 genotypes

**Fig. 3:**
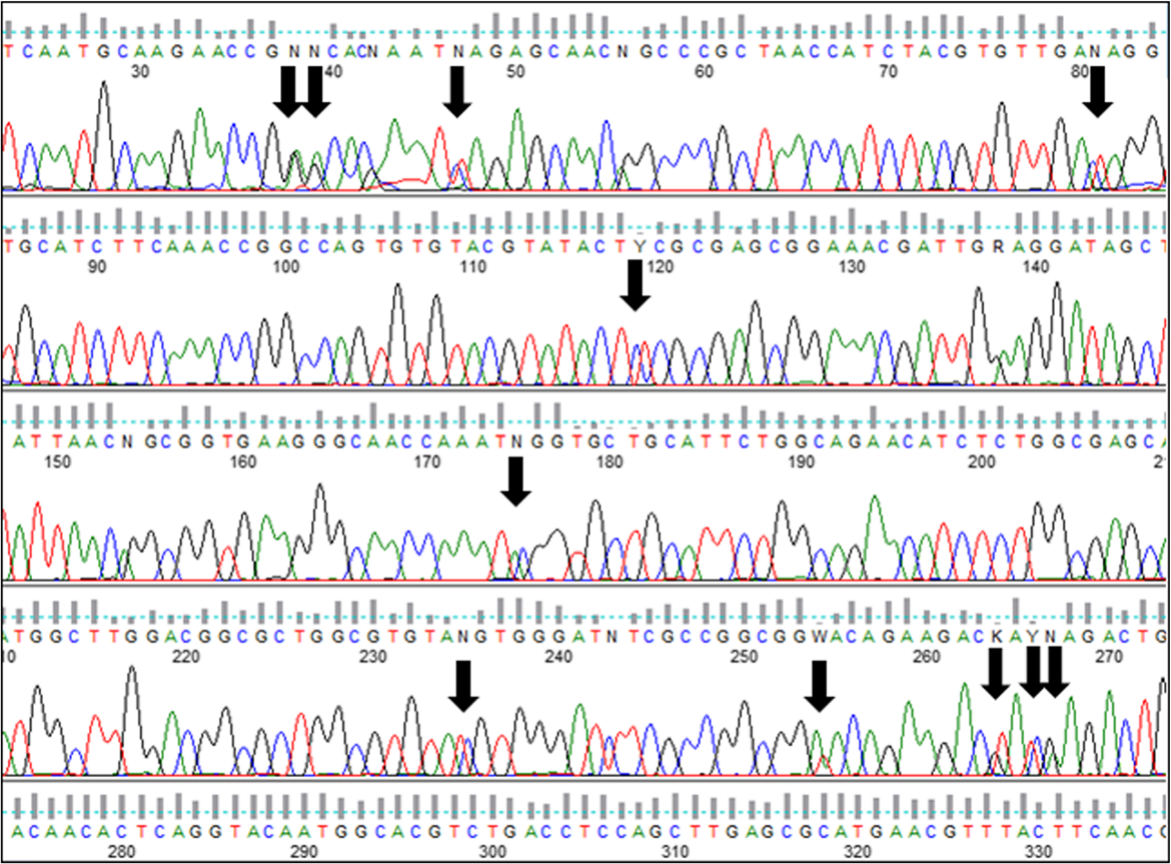
Chromatogram of the β-tubulin–coding gene showing the double peaks due to the presence of different gene copies

All of these strains studied were identified as *Hortaea werneckii* based on ITS sequences and the closest GenBank hits. The ITS sequences were of good quality, whereby the chromatograms had clear peaks and no background noise, with a single exception (EXF-11537), which had three ambiguous positions in both strands. The variant calling for ITS and large subunit (LSU) regions for the strains with sequenced genomes showed that a single genome contained a single variant of the ITS and D1/D2 rDNA sequence (Additional file 1: Table S1). In the D1/D2 domains of 28S rDNA, 10 genotypes (A–J) were recognised with the manual inspection of the sequence alignment, as also seen in the UPGMA analysis (Fig. 1). Within this dataset, eight variable sites were identified, which constituted 1.5% dissimilarity in the 521-nucleotide-long alignment (Table 2). The ITS rDNA sequence alignment was 457 nucleotides long, and the number of variable nucleotides was 23 (dissimilarity 5%), defining 17 different genotypes (Table 3). UPGMA analysis of the 28S rDNA alignment fully reflected the observed genotypes (Fig. 1), but in the ITS rDNA analysis (Fig. 2), the genotypes D/7 (EXF-647) and D/8 (EXF-161, EXF-2788, EXF-96, EXF-631, EXF-9, EXF-10830, EXF-2783) were intermixed with genotype B/7, despite single nucleotide differences. On the other hand, according to D1/D2, the *H. werneckii* ex-type strain (CBS 107.67; EXF-151) formed a single strain genotype J, but for the ITS sequence it belonged to genotype 1. The phylogenetic analyses of the same datasets (i.e. ITS, D1/D2 28S rDNA) with maximum parsimony resulted in overall poorly supported clades (data not shown). Several genotypes showed paraphyletic relationships and not monophyletic groups. Although analyses of the household genes *BTB* and *MCM7* resulted in overall good quality of the sequences, at specific nucleotide positions in the chromatograms for the majority of the strains, double peaks were found in both strands, which led to ambiguity (Fig. 3). On the other hand, unambiguous sequences of both loci were obtained by Sanger sequencing for a number of the strains. The situation was not necessarily the same at both loci, as different strains had, for example, ambiguous sequences in *MCM7*, but unambiguous sequences in *BTB*.

**Table 2:**
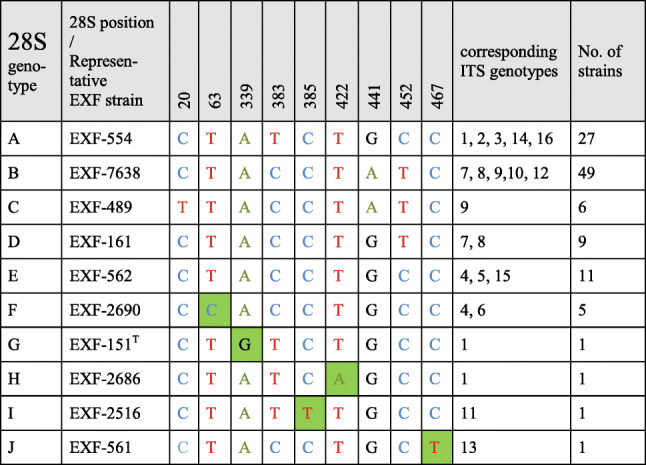
Variable sites in *Hortaea werneckii* alignment of partial D1/D2 28S rDNA sequences

Legend: Green marked nucleotides represent nucleotide change in a single group or strain

**Table 3:**
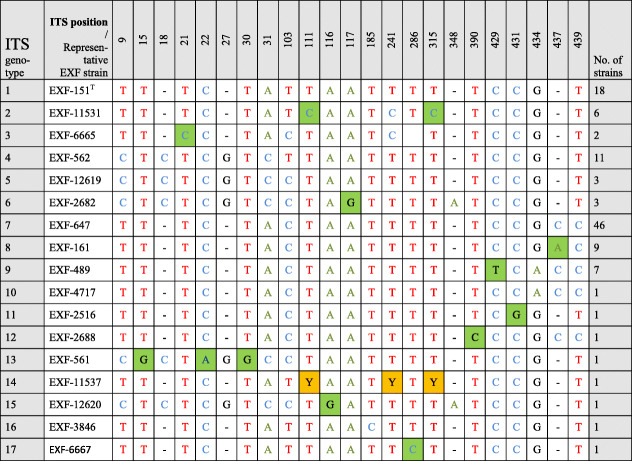
Variable sites in *Hortaea werneckii* alignment of partial ITS rDNA sequences

Legend: Green marked nucleotides represent nucleotide change in a single group or strain. Yellow marked nucleotides (Y) represent ambiguous peaks in the sequence, being either Cytosine (C) or Thymine (T)

**Fig. 4:**
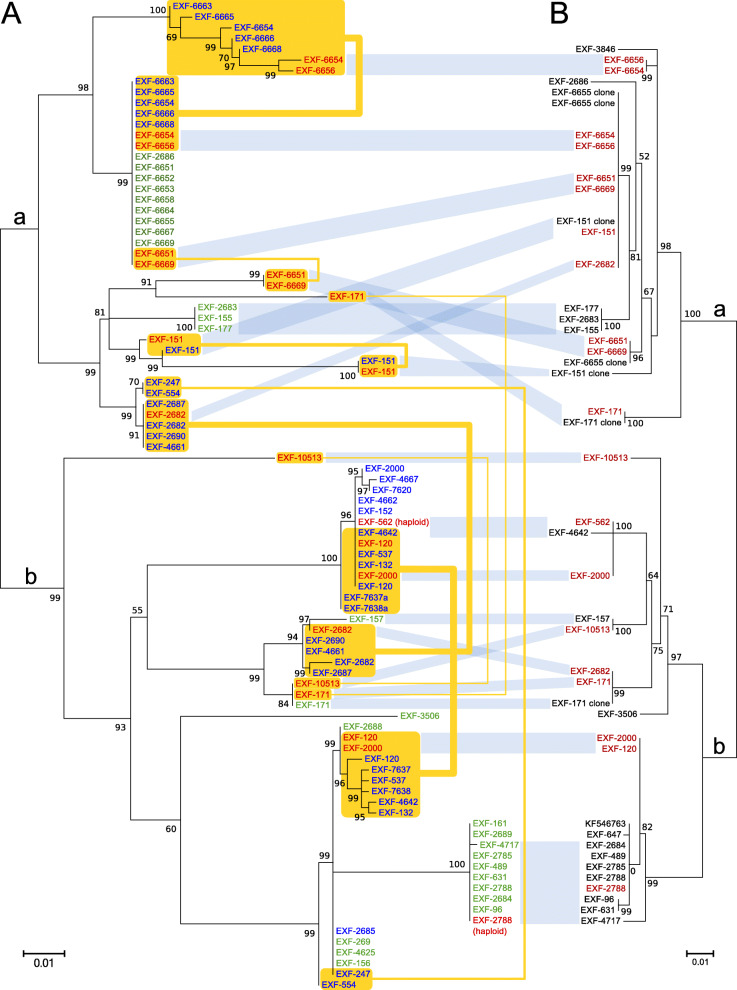
Maximum likelihood phylogenies based on the *MCM7* and *BTB* genes. **a** The *MCM7* gene. The amplicons were amplified by PCR and sequenced by Sanger sequencing. Sequence names without ambiguous nucleotides after sequencing are shown in green. Haplotype estimation was used to separate haplotypes from sequences with ambiguous nucleotides (in blue). Estimated haplotypes with identical phylogenetic positions were collapsed into one sequence. Haplotype sequences recovered from the whole-genome sequences are included for comparison (in red). Haplotype pairs from the same genomes are connected by yellow lines. **b** The *BTB* gene. Only sequences without ambiguous nucleotides and sequences obtained by cloning the amplicons and sequencing individual clones were included in the analysis. Haplotype sequences recovered from the whole-genome sequences are included for comparison (in red). The large phylogenetic clusters observed in both the *MCM7* and *BTB* phylogenies are indicated with ‘a’ and ‘b’

For the *MCM7* gene, these ambiguous sequences were used for computational estimation of the haplotypes, and the estimated haplotypes in most cases matched well to known haplotypes recovered by whole-genome sequencing. Haplotypes from the same strain were positioned in different parts of the intraspecific phylogeny (Fig. 4). The estimated haplotype pairs of strains EXF-2000 (=EXF-225), EXF-4667, EXF-7620, EXF-4662, EXF-152 and EXF-2685 were nearly identical: they had the same phylogenetic position and were collapsed into one leaf per strain in the final tree. Twenty-eight sequences did not contain any ambiguous nucleotide positions. Large phylogenetic clusters largely corresponded to clusters of *MCM7* genes (Fig. 4, clusters marked ‘a’ and ‘b’).

**Fig. 5:**
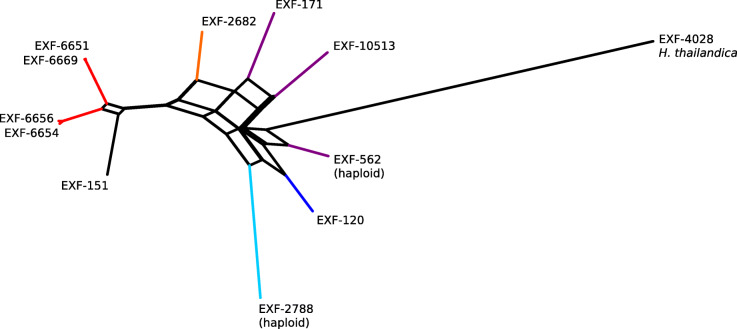
Phylogenetic network of the *Hortaea werneckii* strains with sequenced genomes. The network was reconstructed with the Neighbour-Net algorithm based on the dissimilarity distance matrix calculated from the single nucleotide polymorphism data

The distances between the strains calculated from single nucleotide polymorphism data and used for the construction of the phylogenetic network (Fig. 5) in general matched the distances estimated using the ITS and D1/D2 rDNA sequences. However, the analysis also showed a substantial reticulation within the phylogenetic history of the strains.

### Halotolerance

Halotolerance was expressed as colony diameters of strains on solid MEA media, without NaCl and supplemented with 10, 20, 25 and 30% (w/v) NaCl. This was measured after 9 weeks of incubation, and the data are given in Additional file 1: Table S2. All of the strains grew without NaCl and at up to 20% NaCl within these 9 weeks, with the exception of two strains (i.e., EXF-3846, EXF-2687), which did not grow at 20% NaCl.

**Fig. 6:**
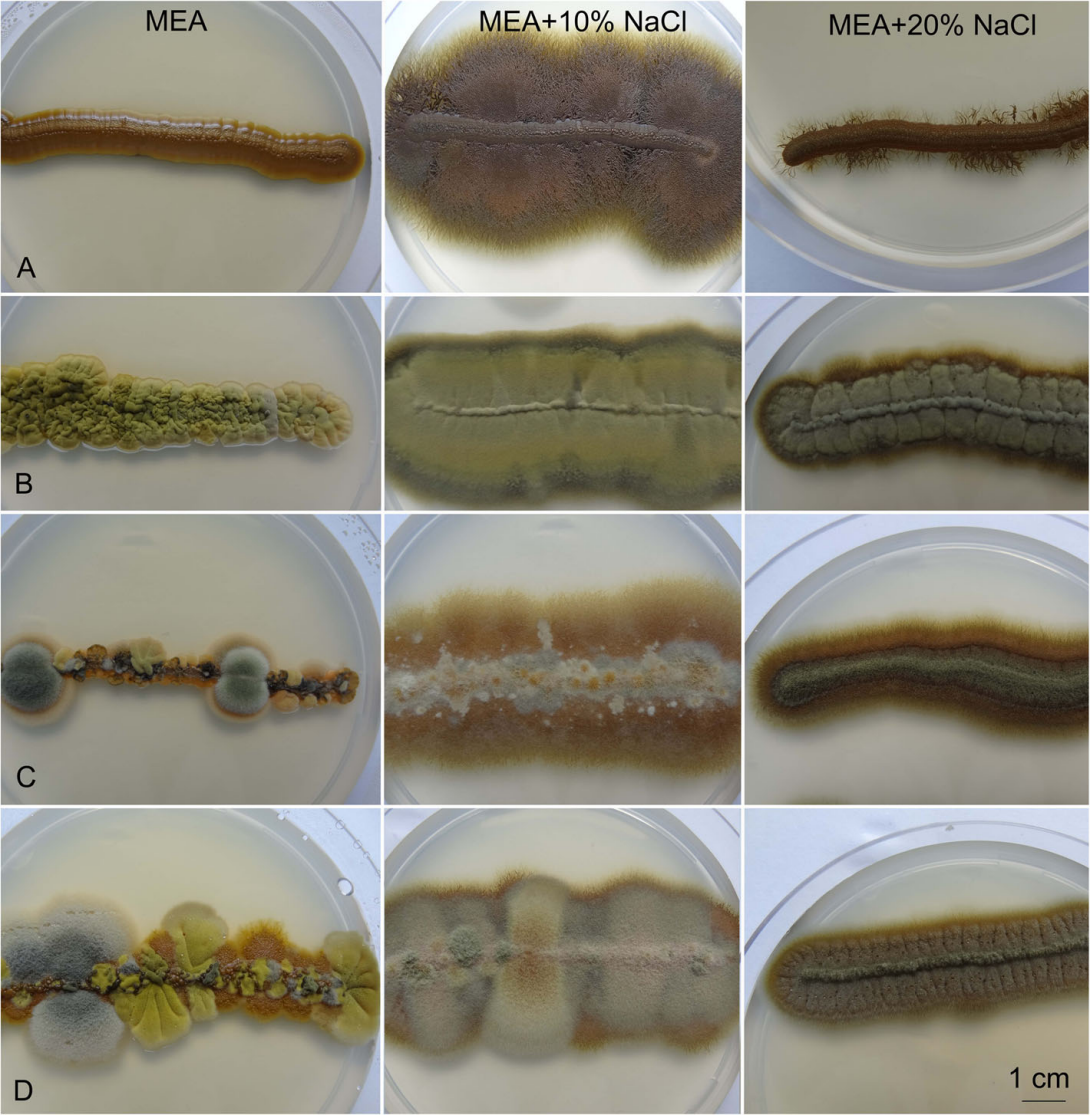
Morphology of *Hortaea werneckii* cultures on malt extract agar (MEA) without and with added NaCl. Left: MEA; middle: MEA + 10% NaCl; right: MEA + 20% NaCl. **a** EXF-120. **b** EXF-4717. **c** EXF-467. **d** EXF-96. Bar = 1 cm (for all)

The growth optimum was at 10% NaCl, which was typically seen as filamentous colonies, with yeast-like cells in the central part. EXF-120 grew as yeasts without NaCl and at 20% NaCl, but showed filamentous immersed growth with 10% NaCl. All other strains were either filamentous on all of the media (representative strain EXF-4717), or formed numerous filamentous sectors within initial yeast colonies without NaCl (representative strains EXF-96, EXF-467), or only filamentous growth without sectors on media with NaCl (representative strains EXF-467, EXF-96) (Fig. 6).

Over the 9 weeks of incubation, only a few of the strains (EXF-247, EXF-554, EXF-156, EXF-269, EXF-4667, EXF-489, EXF-647, EXF-3506) grew at 30% NaCl, to produce colonies of 1–2 mm diam. Some additional strains grew at 30% NaCl after longer incubation periods (data not shown). The largest differences identified by SIMPER tests between the genotypes were in the colony diameters on MEA + 20% NaCl, where the group of strains belonging to LSU/ITS genotype E/4 developed colonies that were 4-fold larger than for the other genotypes. Other strains from this group grew more slowly and did not attain such colony sizes.

### Thermotolerance

**Fig. 7:**
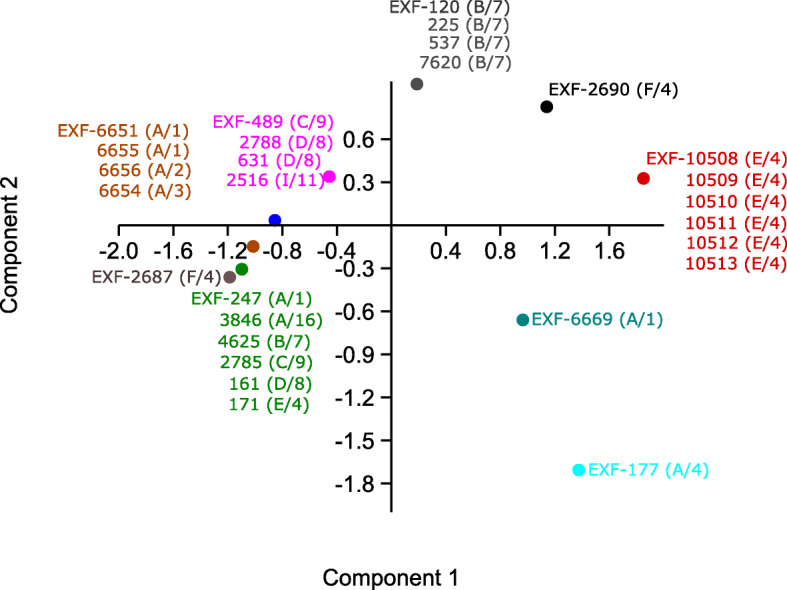
Principal component analysis of *Hortaea werneckii* growth at different temperatures on solid malt extract agar. Strains with the same temperature patterns are coloured the same

The growth of the tested strains on solid MEA media without NaCl and supplemented with 10% NaCl and at different temperatures (5, 15, 25, 37 °C) varied among the strains (Additional file 1: Table S3). Here, some patterns could be correlated with some genotypes, as shown by principal component analysis (Fig. 7). For example, the sea and deep-sea strains from Italy (genotype E/4) grew at 5 °C to 37 °C regardless of the addition of NaCl. Their growth at 5 °C was slow, while it was rapid at 37 °C, and comparable to their growth at 25 °C. However, a human-associated strain (EXF-171) from the same genotype (E/4) did not grow at 5 °C and at 37 °C, which might have been due to its degeneration during subcultivation, as it was originally isolated in 1935. Strains from Atacama, Chile, that belonged to genotypes A/1 to A/3 grew at 5 °C, but only with the addition of 10% NaCl, while they did not grow at 37 °C. The strains assigned to genotype B/7 grew at 15 °C to 25 °C, without and with NaCl, while they grew at 37 °C only with the addition of NaCl. The optimum temperature for all tested strains was at 15 °C to 25 °C.

**Fig. 8:**
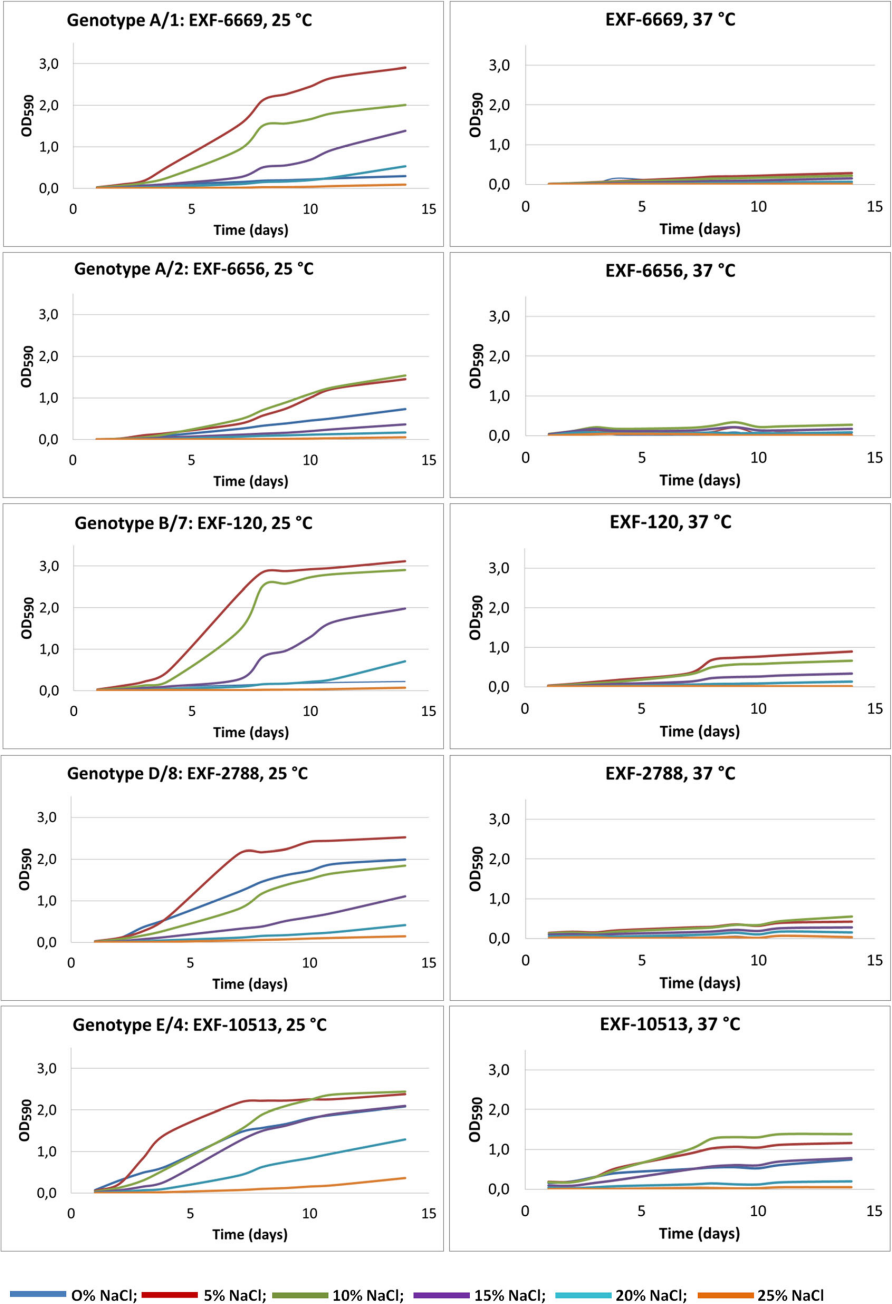
Growth curves of selected *Hortaea werneckii* strains grown in YNB liquid media without and with added 5, 10, 15, 20, and 25% NaCl, incubated at 25 °C and 37 °C

Strains were also tested for temperature tolerance in liquid media (YNB without and with addition of 5, 10, 15, 20, 25% [w/v] NaCl). All of these strains grew faster at 25 °C, and grew at 37 °C with the addition of at least 5% NaCl to the medium (Fig. 8), although they reached lower optical densities at this higher temperature (37 °C, OD_590_ 0.3; 25 °C, OD_590_ 3.0). In agreement with the growth tests on solid media, strain EXF-10813 from the deep sea grew well at 37 °C even without NaCl, although it grew faster with the addition of NaCl (OD_590_ 1.5).

### Morphological studies

**Fig. 9:**
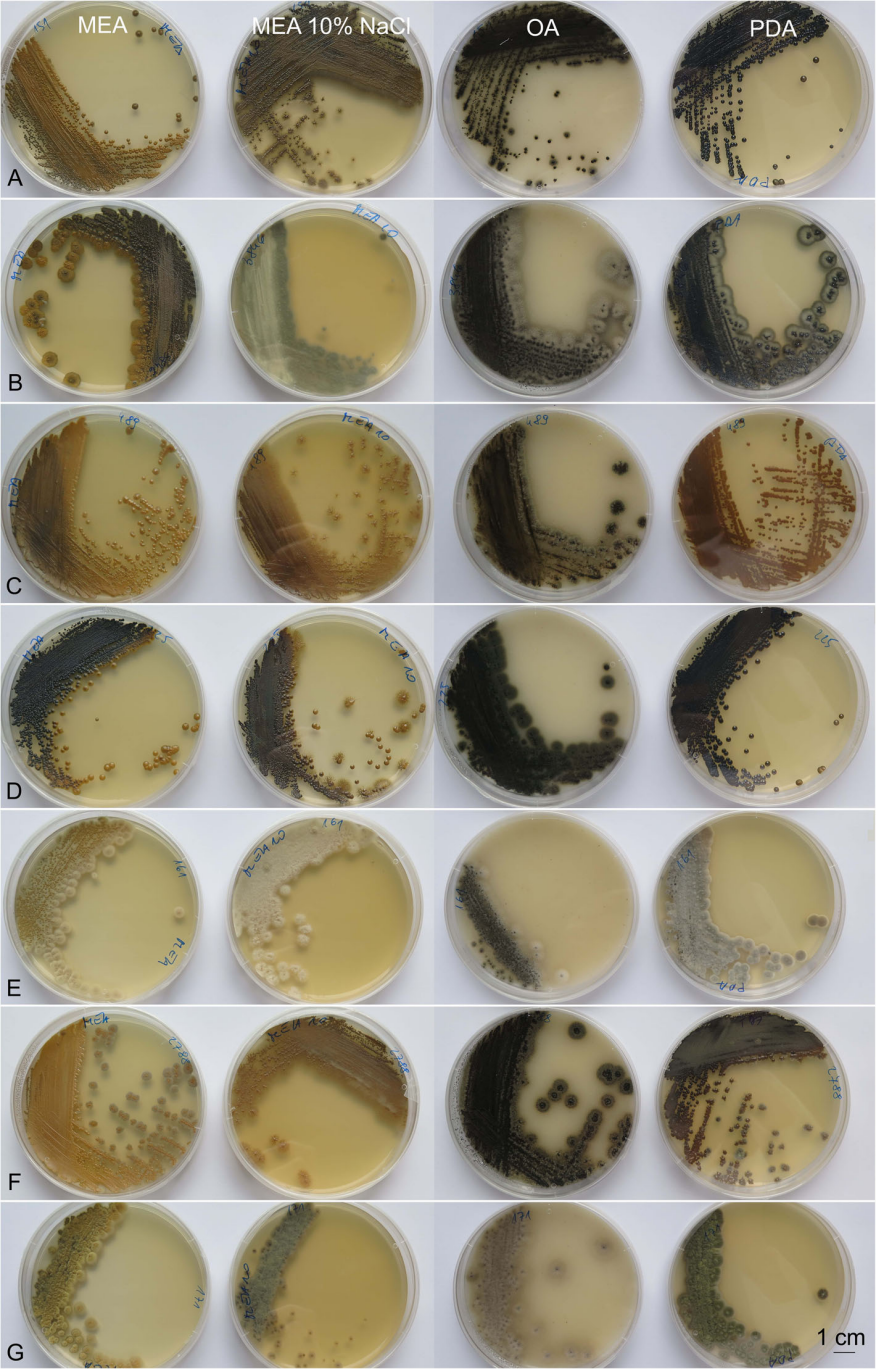
Macromorphology of *Hortaea werneckii* strains grown on malt extract agar (MEA; column 1), MEA + 10% NaCl (column 2), oatmeal agar (column 3) and potato dextrose agar (column 4), for 14 days at 25 °C. **a** EXF-151^T^. **b** EXF-3846. **c** EXF-489. **d** EXF-225. **e** EXF-161. **f** EXF-2788. **g** EXF-171. Bar = 1 cm (for all)

**Fig. 10:**
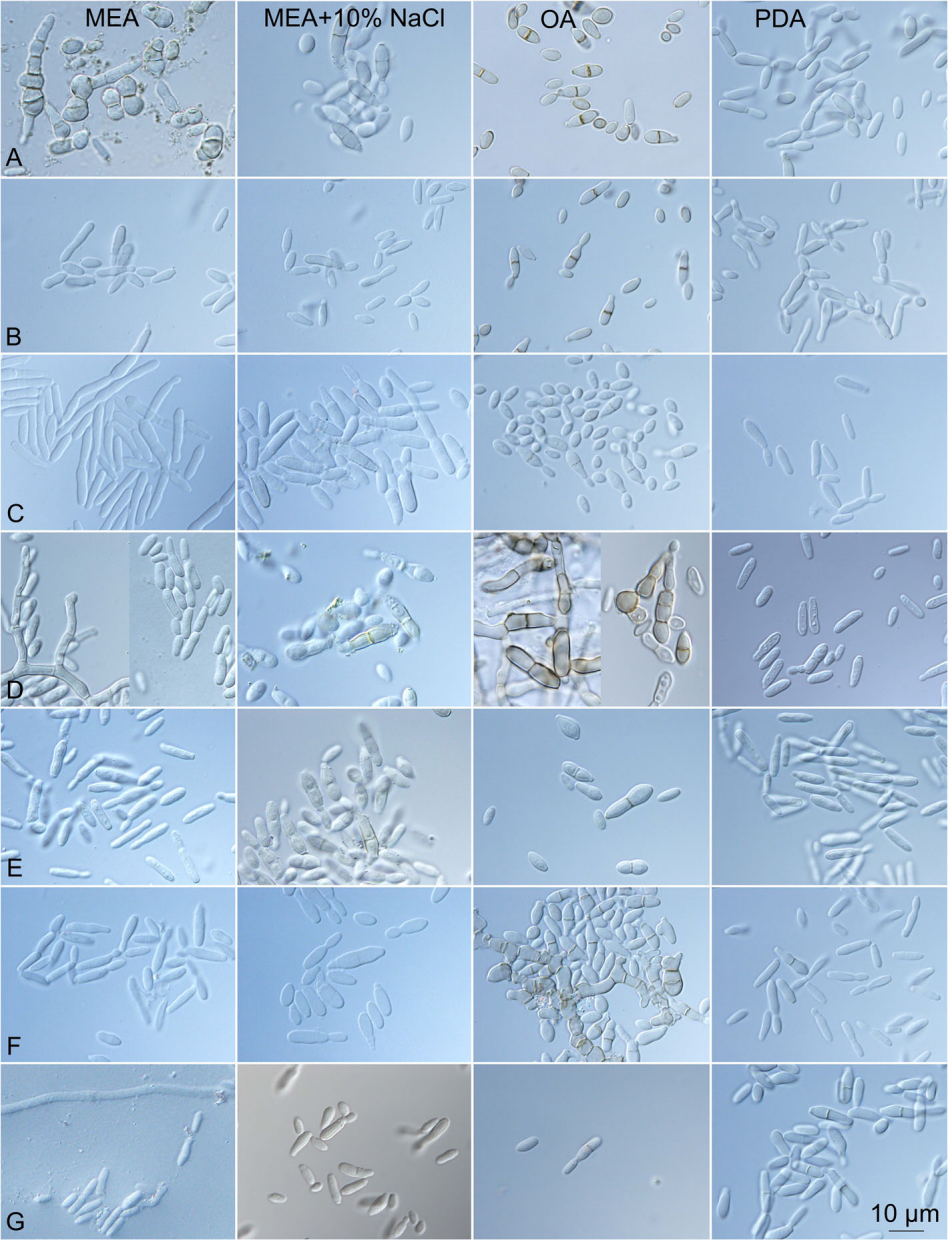
Micromorphology of *Hortaea werneckii* strains grown on malt extract agar (MEA) (column 1), MEA + 10% NaCl (column 2), oatmeal agar (column 3) and potato dextrose agar (column 4) for 7 days at 25 °C. **a** EXF-151^T^. **b** EXF-3846. **c** EXF-489. **d** EXF-225. **e** EXF-161. **f** EXF-2788. **g** EXF-171. Bar = 10 μm (for all)

The colony morphologies differed substantially among strains and across the culture media. Among 34 strains grown for 2 weeks on MEA, nine different colony morphologies were recorded, which differed in terms of colour, structure and size (Additional file 1: Table S4). The colony colours (according to the *Methuen Handbook of Colour*) were most often olive-brown (4E7), but also honey-yellow (5D6), mustard-brown (5E6), yellowish grey (2B2), olive yellow (2D7) and blond greyish yellow (4C4). Colony diameters were 2–7 mm, as yeast-like, filamentous, or mixtures of both forms (Figs. 9 and 10, columns A).

In comparison to MEA, the colonies on MEA + 10% NaCl were initially yeast-like, but developed marginal immersed budding mycelium that were visible as filamentous growth, and some became exclusively filamentous (EXF-6656, EXF-3846), while some appeared filamentous regardless of their growth medium (EXF-161, EXF-171), and reached diameters from 0.5–10 mm. The colony colours here were most often olive-brown (4D, E, F), or olive (3E, F4) to golden brown, to yellowish brown (5D, E), dull green (30D3), or even yellowish grey (4B2).

On OA media, the cultures were dark green (30F5) to black, and reached diameters of 2.5–13.0 mm. The different colonies of the same strain were mostly similar, and were composed of a yeast-like small centre that was surrounded by a wider superficial aerial filamentous margin, although in some cases yeast and yeast/ filamentous colonies developed from a single strain on the same agar plate.

The colonies on PDA were mostly yeast-like and shiny, and reached diameters of 1–9 mm in 14 days. These were yellowish brown (5F7) to olive-brown (4F4–6), with many darker, or they were coloured similar to those grown on OA (e.g. EXF-161).

**Fig. 11:**
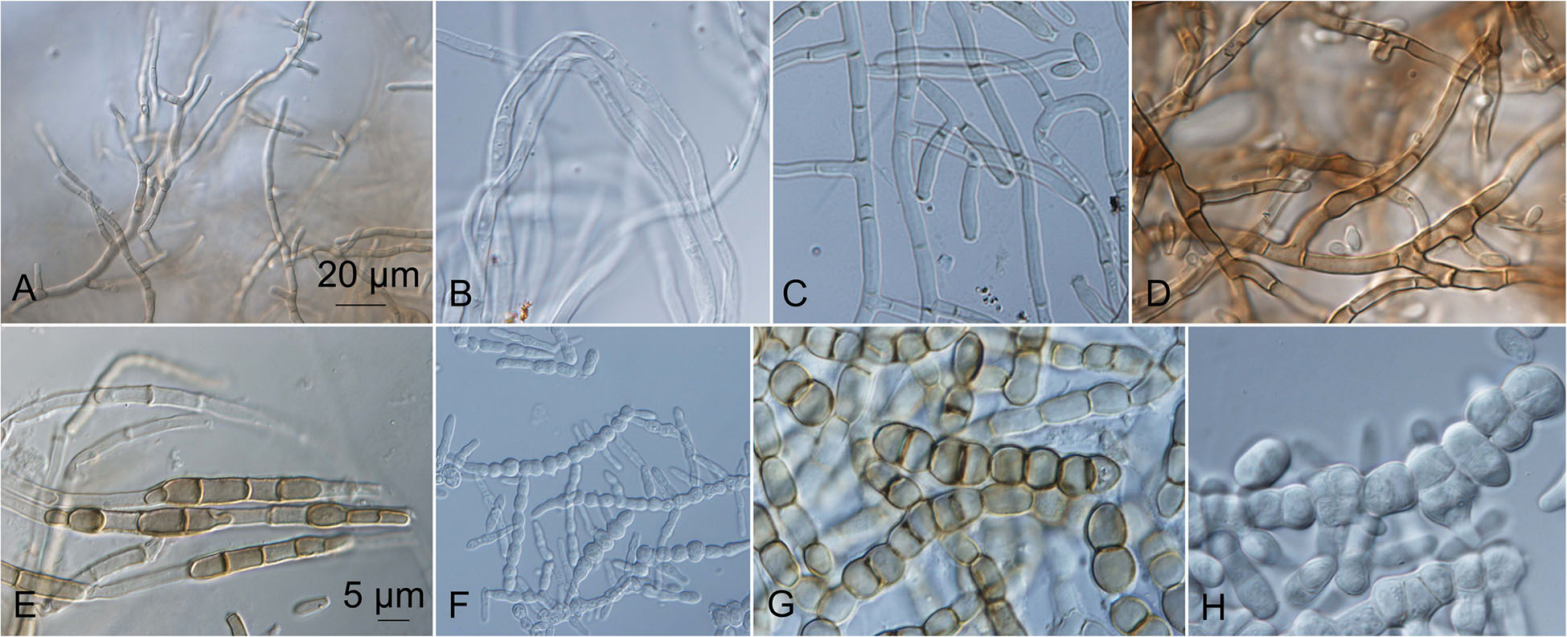
Different kinds of non-sporulating mycelium produced by *Hortaea werneckii* cultures after 14 days at 25 °C. **a** EXF-561: PDA, immersed margin. **b** EXF-161: oatmeal agar (OA), aerial margin. **c** EXF-171: potato dextrose agar (PDA), aerial. **d** EXF-4493: malt extract agar (MEA), immersed centre. **e** EXF-11537: OA, immersed margin. **f** EXF-2682: PDA, centre. **g** EXF-2690: OA, margin. **h**. EXF-2682: MEA + 10% NaCl, centre. Bar = 20 μm (**a**, **f**), 5 μm (**b-e**, **g-h**)

The informations on their microscopic structures on these culture media are shown in Fig. 10. In general, the conidial size, shape, and pigmentation depended on the culture medium, and varied among the strains. On all of the culture media, the colonies started to grow as yeasts, and many of them later developed superficial, aerial, or immersed mycelia. The different kinds of hyphae measured from 2.5–15.0 μm diam. The thin hyphae were either composed of long cells (length, 5–15 μm), or were seen as pseudomycelia that were composed of concatenated two-celled conidia that were constricted at the septa (width, 7 μm). The thick hyphae were additionally longitudinally septate (Fig. 11). The mycelia had lateral proliferating openings that were either almost sessile or developed short single conidiogenous cells, or even complex conidiophores that developed into synnema-like structures.

**Fig. 12:**
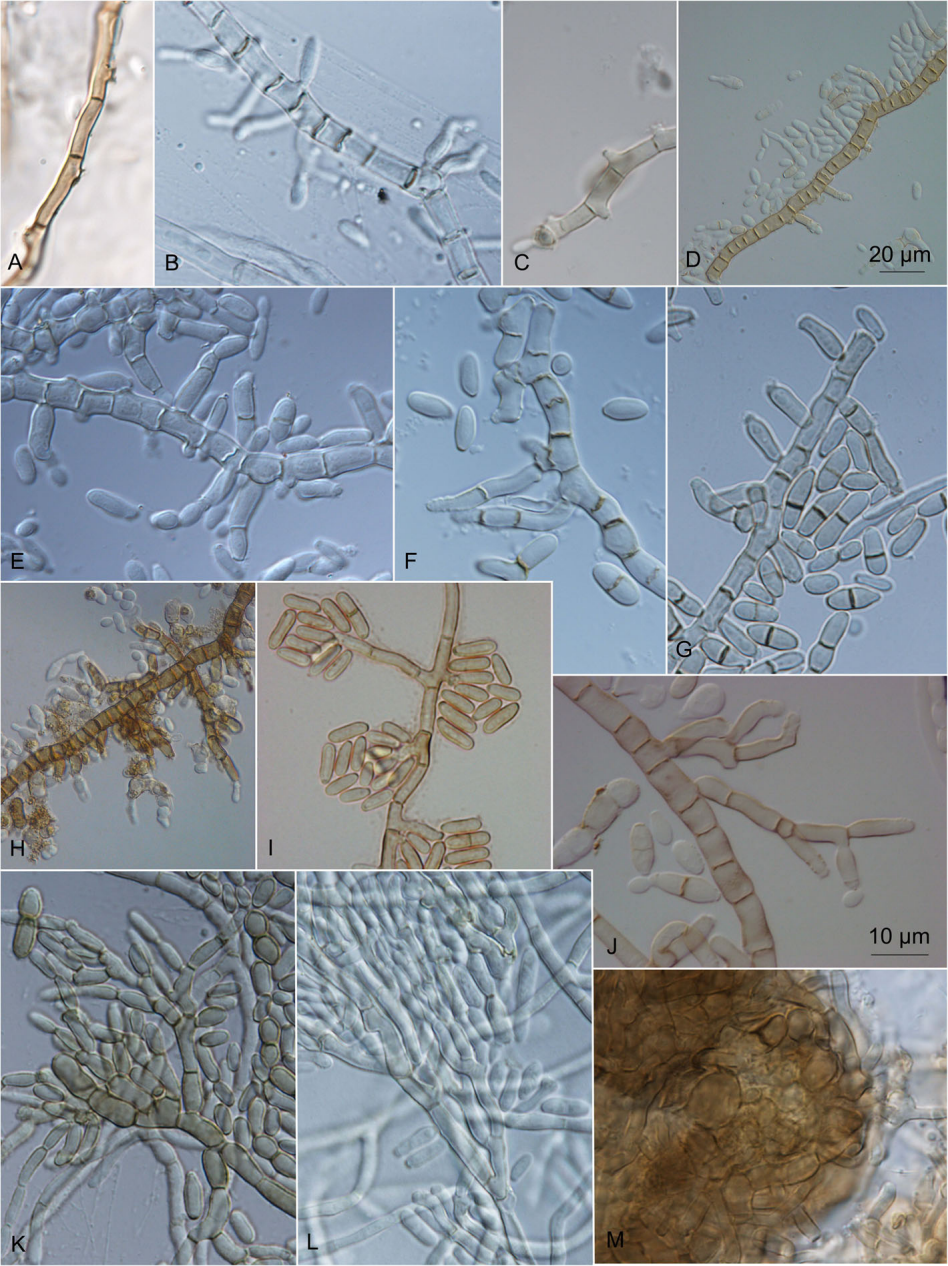
Sporulating mycelium of *Hortaea werneckii* strains in cultures after 14 days at 25 °C. **a** EXF-4493: malt extract agar (MEA), centre. **b** EXF-2682: oatmeal agar (OA), margin. **c** EXF-489: OA. **d** EXF-647: MEA + 20% NaCl. **e** EXF-151: MEA + 10% NaCl. **f** EXF-2687: OA, centre. **g** EXF-171: potato dextrose agar. **h** EXF-6655: MEA + 10% NaCl, margin. **i** EXF-6654: OA. **j** EXF-247: MEA + 10% NaCl. **k** EXF-562: OA, margin. **l** EXF-225: MEA. **m** EXF-2788: OA. Bar = 20 μm (**d**, **i**), 10 μm (**a-c**, **e-h**, **j-m**)

**Fig. 13:**
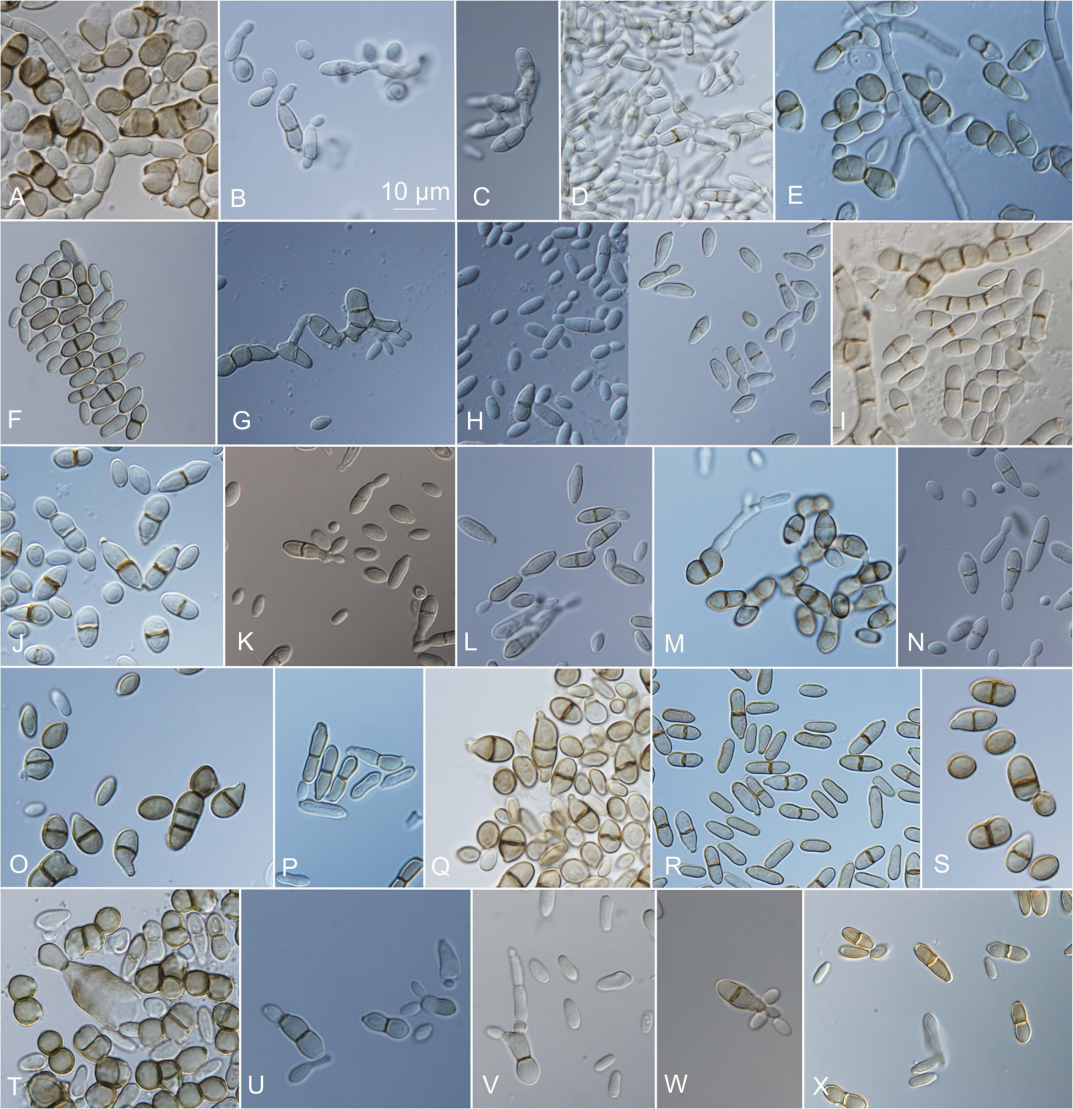
Conidia of *Hortaea werneckii* strains grown on oatmeal agar (OA) for 14 days at 25 °C. **a** EXF-120. **b** EXF-157. **c** EXF-161. **d** EXF-171. **e** EXF-247. **f** EXF-562. **g** EXF-631. **h** EXF-2516. **i** EXF-2687. **j** EXF-2690. **k** EXF-2785. **l** EXF-2788. **m** EXF-3506. **n** EXF-4625. **o** EXF-6651. **p** EXF-6654. **r** EXF-6656. **s** EXF-6669. **t** EXF-7620. **u** EXF-10508. **v** EXF-10512. **w** EXF-10513. **x** EXF-11537. Bar = 10 μm (for all)

**Fig. 14:**
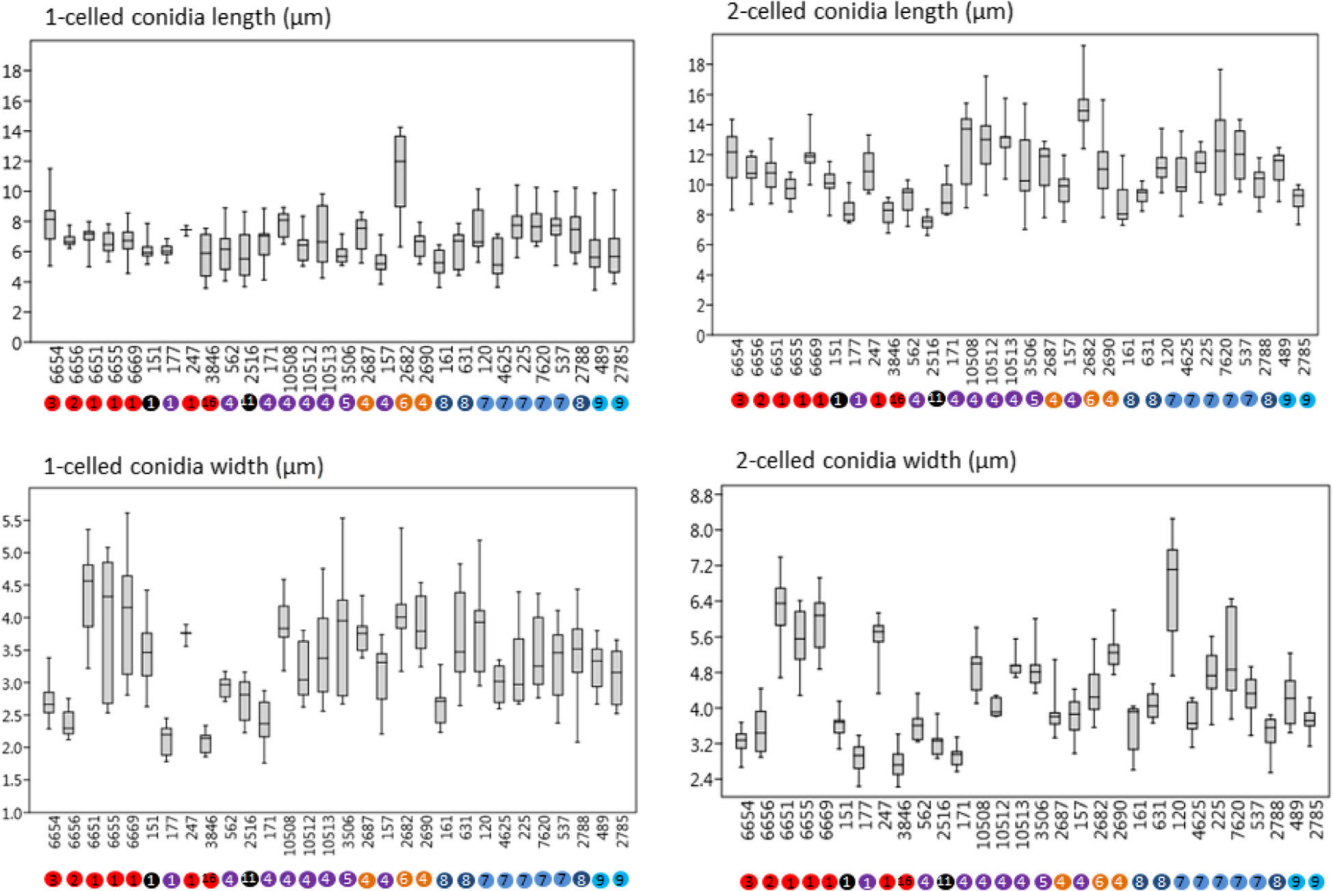
Measurements of the 1-celled and 2-celled conidia of *Hortaea werneckii* strains. Data are means ±standard deviation. 28S rDNA genotypes are indicated as letters (genotype A, red; genotype B, C, D, blue; genotype E, violet; genotype F, orange; miscellaneous LSU genotypes, black), and ITS genotypes as numbers 1–16

On the OA medium, even hyphal clusters that resembled sterile ‘pseudo’ fruiting bodies were seen (Fig. 12). Conidia developed in annellidic conidiogenesis, and were typically one or two-celled and melanized, with bi-polar budding (Fig. 13). On the OA medium they differed in size and pigmentation. One-celled conidia from the colony centres measured 5.0–8.0 × 2.0–4.5 μm, and from the colony margins 5.5–8.0 × 2.0–4.0 μm. Typical two-celled conidia that aggregated in the colony centres as yeast colonies measured 7.5–15.0 × 3.0–6.0 μm, and for some of the strains these were also seen in the marginal parts of the colonies, where they measured 9.0–12.0 × 3.0–8.5 μm (Additional file 1: Table S5). The mean conidial measurements from the centres of the colonies (±standard deviations) are shown in Fig. 14.

**Fig. 15:**
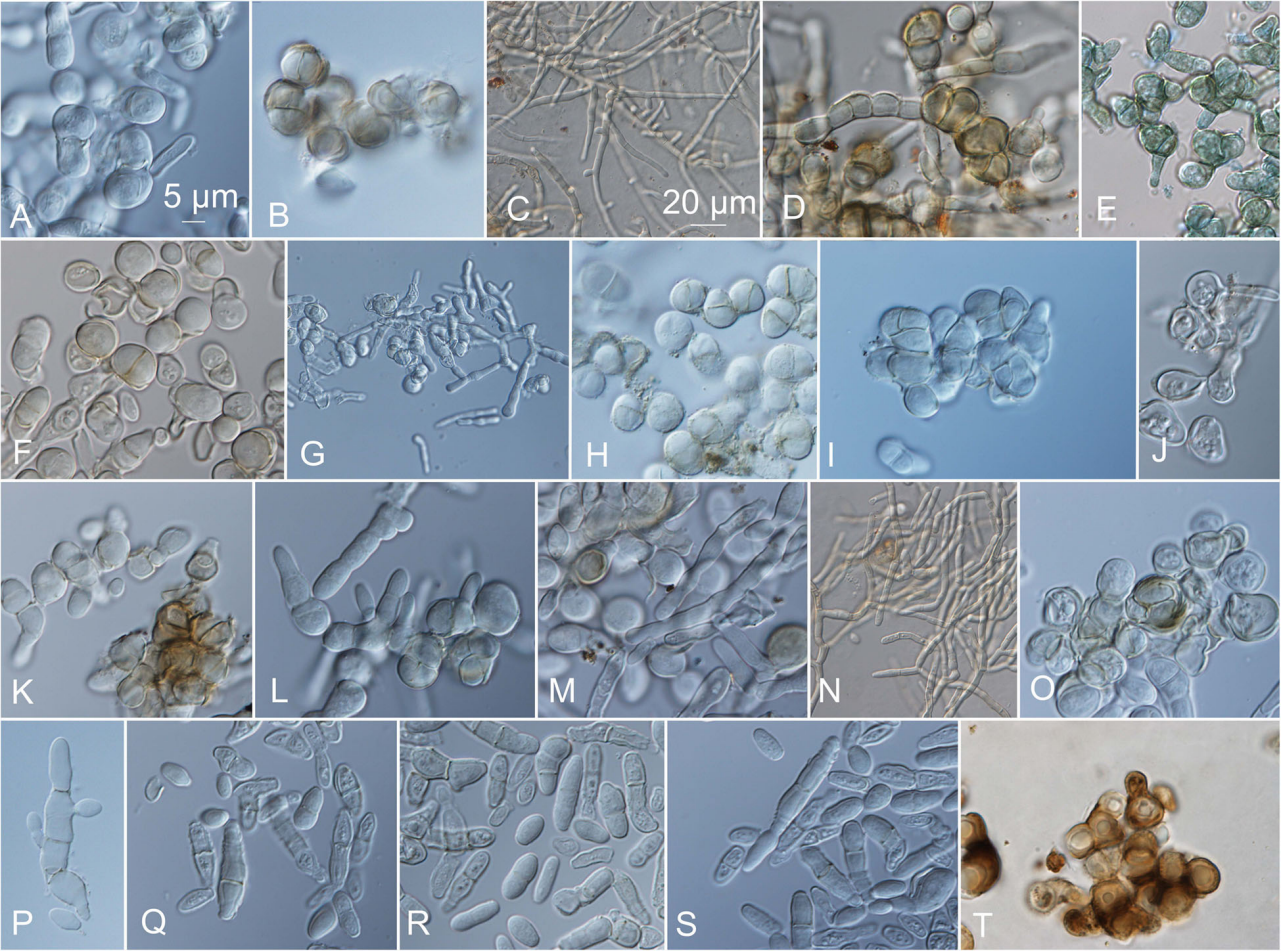
Growth of *Hortaea werneckii* strains after 21 days at 37 °C on solid malt extract agar (MEA) medium supplemented with 10% NaCl (if not indicated otherwise). **a**, **b** EXF-120. **c**, **d** EXF-151^T^. **e** EXF-151^T^ in liquid YNB. **f** EXF-157. **g** EXF-225. **h** EXF-269. **i** EXF-537. **j** EXF-561. **k** EXF-631. **l** EXF-2690. **m** EXF-3506. **n** EXF-6651. **o**, **p** EXF-7620. **q** EXF-10508 (on MEA). **r** EXF-105012 (on MEA). **s** EXF-10513. **t** EXF-11537 (on MEA). Bar =5 μm (**a**, **b**, **d**-**f**, **h**-**m**, **o**-**t**), 20 μm (**c**, **g**, **n**)

Growth in the form of meristematic clusters was evident for almost all of the strains growing on MEA at 37 °C, and this also appeared in the centres of 14-d-old colonies on PDA and MEA, and for some of the strains also on MEA with 10 to 30% NaCl (Fig. 15).

### Use of carbon and nitrogen sources

The 190 carbon assimilation tests were divided into 10 groups according to their main roles in fungal metabolism: pentose catabolic pathway; galactose pathway; starch and sucrose metabolism; uronic acid metabolism; glycolysis and branches; pyruvate metabolism; citrate cycle; amino-acid and protein metabolism; compatible solutes; and aromatic pathways. An additional group of ‘other carbon sources’ was as defined previously by Nai et al. (2013). Some substrates involved in more than one metabolic pathway were classified into two groups (e.g. sugar alcohols, with D-sorbitol, D-arabitol and L-arabitol classified both as the pentose catabolic pathway and as metabolism of compatible solutes).

Of the five strains tested, good growth was detected in 52 of the carbon assimilation tests (27.4%) that defined the pentose catabolic pathway, galactose pathway, starch and sucrose metabolism, glycolysis and branches, pyruvate metabolism, citrate cycle, compatible solutes, uronic acid metabolism, and other carbon sources. The most efficiently catabolised substrates were those from the pentose (D-ribose, D-xylose, L-arabinose, L-arabitol) and galactose (D-galactose, D-melibiose, D-raffinose) catabolic pathways, glycolysis and branches (D-glucose, D-mannose, arbutin, gentiobiose, laminarin), and metabolism of compatible solutes (D-mannitol, D-trehalose). None of the strains catabolised 46 of the substrates (24.2%), a large proportion of which belonged to amino-acid and protein metabolism (34.8%; 16/46) and other carbon sources (32.6%; 15/46). The use of the remaining amino-acid and protein metabolism sources and the aromatic pathways varied between the strains, and ranged from good to weak, and negative (see Additional file 1: Table S6 for details).

The catabolism of nitrogen was tested on 95 nitrogen sources that were divided into eight groups: simple nitrogen sources; amino acids and derivates; primary amines; amides; amino sugars; nucleobases and derivates; aminated alkenes; and dipeptides. The strains grew well on the majority of these nitrogen substrates (from 68 to 90% of the tests), except for EXF-2788, which grew well only on 31 (32.6%) of these nitrogen substrates, and did not grow on 40 (42.1%) of them (Additional file 1: Table S7). Also, the SIMPER test revealed that the lack of catabolism of numerous nitrogen sources by this strain (e.g. methylamine, D-glutamic acid, L-histidine, N-butylamine, uric acid, D-alanine, ethylamine, cytosine, cytidine, D,L-α-amino-N-butyric acid, L-methionine, uridine) contributed the most to its separation from the remaining strains. All of the strains showed little or no catabolism of amino-acid D-lysine, primary amine ethylenediamin, amino sugars N-acetyl-D-mannosamine and N-acetyl-D-galactosamine, and nucleobases thymidine, thymine and uracil.

### Enzymatic tests

The results of the enzymatic tests are given in Additional file 1: Table S8. The proteolytic activities on casein were positive or weak for around 60% of the strains, and became weaker or were lost with addition of 5 and 10% NaCl at 25 °C; at 37 °C, only 30% of the strains were positive. The proteolytic activities on gelatine were generally positive and became stronger with the addition of NaCl. The esterase activities were positive for all of the strains at 25 °C, even in the presence of 5 and 10% NaCl, while at 37 °C, addition of 10% NaCl (but not 5% NaCl) largely inhibited the esterase activity.

## DISCUSSION

Black yeasts are a polyphyletic group of melanized polymorphic fungi that can grow in yeast-like and filamentous forms. They can express an ‘extremophilic ecotype’ (Gunde-Cimerman et al. 2000) that is characterised by thick melanized cell walls, slow, often meristematic growth, and proliferation with endoconidiation. Globally, black yeasts populate different extreme environments, from hypersaline coastal salterns worldwide (Gunde-Cimerman & Zalar 2014), to surfaces and subsurfaces of rocks at high or low temperatures (Wollenzien et al. 1995, Selbmann et al. 2005) and Arctic glacial ice (Gunde-Cimerman et al. 2003). Many are involved in opportunistic infections, which demonstrates a certain coherence between extremotolerance and opportunism (de Hoog et al. 2005, Teixeira et al. 2017)*.*

*Hortaea werneckii* is a cosmopolitan, extremely halotolerant, black yeast without a known sexual morph. Due to its phenotypic plasticity, historically, *H. werneckii* was placed in numerous genera (e.g. *Cladosporium*, *Exophiala, Dematium, Pullularia, Aureobasidium, Sarcinomyces, Pheoannellomyces*) before being ascribed to a new genus, due to its special method of conidiogenesis (Nishimura & Miyaji 1984), of which it was designated the type species. Two additional, so far rarely isolated, species have been described in the last decade: *H. acidophila* from lignite (Hölker et al. 2004) and acidic saline soils (Hujslova et al. 2010); and *H. thailandica* originally described from plant material in Thailand (Crous et al. 2009), and was later also found in subglacial ice on Svalbard (Zalar & Gunde-Cimerman, unpubl.). However, *H. acidophila* was later transferred to the genus *Neohortaea* in *Capnodiales* based on its phylogeny for nuclear ribosomal and five household genes (Quaedvlieg et al. 2014).

*Hortaea werneckii* is the causative agent of *tinea nigra*, and its primary habitat is hypersaline environments and seawater (Gunde-Cimerman & Zalar 2014). It can also grow in other osmotic environments, as recently demonstrated by its discovery in house dust in Hawaii (Humphries et al. 2017) and on spider webs, walls and sand in arid Atacama caves that have been exposed to marine aerosols (Azúa-Bustos et al. 2010), as indicated above. In the present study, 98 strains were studied that were collected worldwide from natural hypersaline environments and Atacama Desert, and from human, animal and plant sources.

All tested *H. werneckii* strains were halotolerant and grew from 0 to 25% NaCl, and some strains even grew at 30% NaCl (Fig. 6, Additional file 1: Table S2). In agreement with other studies (Gunde-Cimerman et al. 2000, de Hoog et al. 2000, Butinar et al. 2005), these strains grew best from 15 to 25 °C, although some strains grew at 5 °C and 37 °C, without or with addition of at least 5 to 10% NaCl (Fig. 8, Additional file 1: Table S3). This indicated their previously underestimated pathogenic potential (de Hoog et al. 2000).

The morphological data (except for the conidial size) were in agreement with previous descriptions (Nishimura & Miyaji 1984, de Hoog et al. 2000). In particular, the two-celled conidia were larger than stated in the species description (7.0–9.5 × 3.5–4.5 μm) and varied considerably across the different culture media (7.5–15.0 × 2.5–6.5 μm; Additional file 1: Table S5).

The study of the use of carbon and nitrogen sources for five selected *H. werneckii* strains is the most comprehensive study to date (Additional file 1: Tables S6–S7), and the data are in line with previous data obtained by traditional methods (Zalar et al. 1999, de Hoog et al. 2000). These strains showed various carbon and nitrogen assimilation patterns, with the exception of the haploid strain EXF-2788, which did not catabolise numerous nitrogen substrates.

The extracellular enzymatic activities of four *H. werneckii* strains, which were recently evaluated by Formoso et al. (2015) and Elsayed et al. (2016), were dominated by plant degrading enzymes, including amylase, lipase, esterase, pectinase and/or cellulase, and no animal-related enzymes, like albuminase, keratinase, phospholipase and DNAse. All of the tested strains were urease positive, while caseinase, gelatinase and laccase were variable. The data are in agreement with both of the previous studies (Additional file 1: Table S8), although the enzymatic activities varied considerably between the strains.

*H. werneckii* strains show a unique natural ecology and pathogenic potential, along with high phenotypic and genotypic variability. These aspects were indicated previously and are defined further in this extensive study, and they have raised the question whether a species complex might be concerned (Boekhout et al. 1993, Uijthof et al. 1994, de Hoog et al., 1999, Zalar et al. 1999, Cabañes et al. 2012, Formoso et al. 2015, Elsayed et al. 2016, Marchetta et al. 2018). This also prompted our phylogenetic analysis here. All of the previous studies were based on smaller sets of strains (i.e. from three to 67), and used different genotypic markers and methods, like karyotyping, different PCR fingerprinting techniques, and ITS/28S rDNA sequence variability.

In the present study, the 98 worldwide strains were analysed according to alignments of D1/D2 and ITS rDNA sequences. These analyses allowed the definition of 10 and 17 genotypes, respectively, that are based on 1.5 and 5% dissimilarities, respectively (Figs. 1, 2). In the ITS analysis, several phylogenetically non-informative single point mutations were seen, which resulted in numerous paraphyletic groups in the phylogenetic analysis. The majority of strains that belong to genotype A were derived from tropical, subtropical and southern hemisphere hypersaline environments, while genotypes B to E contained more strains from Mediterranean regions. Although the majority of the strains originated from the Mediterranean area, they were spread (with few exceptions) among almost all of the recognized genotypes. Although genotype B included some strains from animals (i.e. fish, corals) and plants, animal-related and plant-related strains were also found in genotype A. The five strains from humans grouped in the 28S/ITS genotypes A/1 (France, unknown), E/4 (Brazil), F/9 (Italy) and G/1 (type strain from Portugal), and not in genotype B. Interestingly, all of the strains from the Atacama Desert grouped into genotype A for 28S rDNA, but based on ITS, they were divided into four genotypes: A/1, A/2, A/3, and A/14. For these strains only, variability was also noted for conidial size: strains of genotype A/1 (EXF-6651, EXF-6655, EXF-6669) had double the conidial width in comparison to strains of genotype A/3 (EXF-6654, EXF-6656) (Figs. 13o-s, 14). Additionally, random representatives of all of the genotypes were highly variable. This intraspecific variability indicates a potentially problematic division into genotypes, as exemplified by the strains associated with *tinea nigra*.

The taxonomy of a potential *H. werneckii* species complex was further resolved by Sanger sequencing of the standard taxonomic markers; i.e. the partial *BTB* and *MCM7* genes. Most of the resulting sequences contained ambiguities (Fig. 3). This problem was initially explained by the finding of genome duplication in the single strain where the genome was sequenced (Lenassi et al. 2013, Sinha et al. 2017). A more recent study by Gostinčar et al. (2018) analysed an additional 11 *H. werneckii* genomes, and showed that the majority of the strains were diploid and highly heterozygous. The large divergences within the estimated *MCM7* haplotype pairs of individual *H. werneckii* strains matched well the proposed formation of hybrids between heterozygous strains of *H. werneckii* – even though comparative genomics indicated that *H. werneckii* is clonal (Gostinčar et al. 2018). In the analysis here of the *MCM7* sequences that included the whole-genome sequence-derived and computationally estimated haplotypes, in three cases the pairs of haplotypes of four or more strains shared the same phylogenetic history (Fig. 4), which might have resulted from the same hybridization event. In addition to this, several such events that involved smaller numbers of strains were indicated. In several cases the phylogenetic distances between the haplotypes were substantial.

The situation shown by the phylograms of the *BTB* and *MCM7* genes depicted in Fig. 4 illustrates the difficulties encountered when investigating intraspecific phylogeny of *H. werneckii* using standard approaches of sequencing molecular markers. In some cases, both copies of the marker genes were amplified. If they did not contain insertions/ deletions, Sanger sequencing produced useful results, although with ambiguous nucleotides in positions that differed between the copies (Fig. 3). After careful investigation of the sequencing chromatograms, the resulting sequence was used for estimation of both haplotypes, a process that was sometimes relatively accurate (e.g. EXF-120), and sometimes unsuccessful (e.g. with the production of two nearly identical haplotypes; such as for EXF-2000/ EXF-225), as seen from the comparisons with the true haplotypes derived from the corresponding whole-genome sequences. In some cases, the sequencing produced good quality sequences with no ambiguities. Theoretically, this can occur if the strain is indeed haploid (e.g. EXF-2788, and possibly other strains in the same cluster), if the two copies of the gene in a diploid genome are identical, or if PCR favours amplification of one copy over the other (e.g. EXF-6669). This last case can lead to an incomplete result – the apparently clear positioning of the species in the intraspecific phylogeny, although the other gene copy might be positioned in a very different part of the gene tree. If both copies are amplified, it is possible to sequence each copy separately by cloning the amplicon and sequencing several clones, as was achieved for β-tubulin (Fig. 4; strain EXF-6655). However, if the amplification is strongly biased towards one of the copies, this approach is not feasible, as all of the sequenced clones will contain the same copy of the gene.

Interestingly, sequencing the ITS and D1/D2 rDNA markers was not hampered by the heterozygosity that resulted in mixed sequences with ambiguous nucleotide positions (Additional file 1: Table S1), as for all of the other genes of *H. werneckii* analysed here. This can be explained by concerted evolution, a process that is responsible for sequence homogenisation of different gene copies in the same genome (Liao 1999). If this is the case, concerted evolution in *H. werneckii* appears to be happening more efficiently in ribosomal sequences than in other genes. A reason for this might be the organisation of the ribosomal DNA in tandem repeats, and the resulting increased opportunity for unequal crossing-over, which has been linked to concerted evolution of the ITS region in fungi (Naidoo et al. 2013). ITS and D1/D2 rDNA phylogenetic markers can thus be used to discern the intraspecific phylogeny of *H. werneckii* without the technical problems that prevent the use of other markers, as discussed above. However, it has to be noted that the phylogeny estimated based on ITS and D1/D2 rDNA markers is a simplified approximation of the real evolutionary history of the species, as it ignores the substantial reticulation of the *H. werneckii* phylogeny (as demonstrated in Fig. 5), which is a consequence of hybridization into persistent and highly heterozygous diploids (Gostinčar et al. 2018).

Hybridization events and changes in ploidy in fungi have been described in other species, but with distinct differences compared to these observations for *H. werneckii*. In *Saccharomyces cerevisiae* a whole genome duplication event occurred through an ancient hybridization, which was then followed by loss of most of the duplicated regions (Marcet-Houben & Gabaldón 2015). Apart from this, variations in ploidy and aneuploidy are relatively frequent and are thought to be important for rapid adaptation to environmental changes (Sunshine et al. 2015). Interspecies hybrids have been described in fungi as well, and while most of these are in species only known as asexual morphs, *Zygosaccharomyces parabailii* was shown to have regained fertility by inactivation of one mating-type locus (Ortiz-Merino et al. 2017). Another well-studied example is *Candida albicans*, a predominantly diploid species that nevertheless has a high degree of genome plasticity, where frequent losses of heterozygosity and gross chromosomal rearrangements might result in aneuploidy (McManus & Coleman 2014). Although reproduction in *C. albicans* is predominantly clonal, this species can also use a parasexual cycle that involves the formation of tetraploid progeny from the mating of diploid parents, the former of which subsequently revert to diploidy by concerted chromosome loss (Bennett & Johnson 2003). While double peaks on sequencing chromatograms can be easily dismissed as a result of suboptimal sequencing quality, they have been reported in at least one other case – in the yeast genus *Ogataea*. Here, such double peaks were reported to be an indication of heterozygous diploidy or of the existence of gene paralogues that were both/all amplified with the primer sequences used (Čadež et al. 2013).

The failure to recognise hybrids is not only a danger in terms of serious taxonomic misunderstandings, but can also have implications beyond taxonomy. Inderbitzin et al. (2011a) indicated such implications in the management of pathogenic fungi. In this field, the existence of hybrids was first inferred from credible morphological and genetic evidence, and later based on molecular phylogenetics (Inderbitzin et al. 2011a). Hybrids are known in basidiomycete rust genera (e.g. *Tilletia, Melampsora, Cronartium, Puccinia*), ascomycetous plant pathogens (e.g. *Botrytis*, *Fusarium*, *Ophiostoma*, *Verticillium*), yeasts (e.g. *Saccharomyces, Candida*, *Cryptococcus*), and endophytic fungi. Hybridizations or recombination of introduced fungal pathogens with related resident fungi often results in strains that can infect an expanded range of hosts (Brasier, 2000). By whole genome sequencing of two strains, Fogelqvist et al. (2018) confirmed the findings of Inderbitzin et al. (2011b) for *Verticillium longisporum*. Their comparatively large draft genomes compared to other ascomycete fungi studied were composed of two parts, where one lineage was more ancient than the part that was more closely related to *V. dahliae*.

The pattern of intraspecific hybridization seen here in *H. werneckii*, which is otherwise a clonal species, represents an unusual strategy that to the best of our knowledge has not been described in other fungi to date. Enriching the genomic toolbox with two different copies of the same gene, or combining traits that are otherwise combined via sexual reproduction, might promote survival under extreme environmental conditions. However, the possible benefits of heterozygous diploid genomes for the survival of *H. werneckii* remain to be investigated. The presence of four ITS genotypes in a small area was seen for the Atacama cave strains, where some diploid strains were almost identical in one haploid subgenome, but differed substantially in the other haploid subgenome (Gostinčar et al. 2018). This makes *H. werneckii* an intriguing extremotolerant experimental system with persistent stable haploid and diploid strains and little, if any, noise introduced by sexual recombination.

## CONCLUSIONS

Ongoing recent trends in fungal taxonomy that are supported by powerful phylogenetic multi-locus analyses encourage descriptions of new species, which can often be based on only a single strain, or a few strains, and/or a few nucleotide differences. When using a limited set of strains, the decisions to recognise cryptic species have to be taken with great caution, as these analyses can be seriously misleading. The present study that is based on numerous geographically and time-spread strains is characterised by a highly plastic and unusual fungal species that challenges this concept. In the majority of these *H. werneckii* strains, on the one hand, there were ambiguous positions for the household genes routinely used in taxonomic studies, and on the other hand, there were the unambiguous sequences of the ribosomal genes. According to our estimates, around 20% of these *H. werneckii* strains were haploid, while the others were diploid. This situation prevented standard species delineation and requires a restrained and adjusted taxonomic approach. Analysis of a smaller number of geographically separated strains or a restricted number of phenotypic and clustering analyses might have resulted in the description of several new species. We believe that whenever there is the need to deal with extremotolerant and (or) phenotypically variable fungi, as exemplified by many black yeasts, a tailor-made rather than a standard approach to species recognition should be considered.

## Additional file

Additional file 1: **Table S1.** Genome mining for ITS and D1/D2 rDNA sequences. The number of reads with a sequence identical to the reference sequence of the strain EXF-225 (ref.) and number of reads with an alternative sequence (alt) are shown. **Table S2.** Halotolerance of the *Hortaea werneckii* strains over 9 weeks on malt extract agar without and with added 5, 10, 15, 20, 25, and 30% NaCl. **Table S3.** Growth of the *Hortaea werneckii* at different temperatures (5, 15, 25, 37 °C) on solid malt extract agar without and with added 10% NaCl. **Table S4.** Growth of the *Hortaea werneckii* strains on the different culture media. **Table S5.** Conidia and hyphae measurements for the *Hortaea werneckii* strains after 2 weeks of incubation on oatmeal agar at 25 °C. **Table S6.** Carbon and nitrogen assimilation for the *Hortaea werneckii* strains. **Table S7.** Nitrogen assimilation for the *Hortaea werneckii* strains. **Table S8.** Proteolytic (casein, gelatine) and lipolytic (esterase) enzymes of the *Hortaea werneckii* strains grown on solid media at 25 °C and 37 °C, and with addition of 0, 5, and 10% NaCl. (XLSX 64 kb)

## Abbreviations

*BTB*: Partial genes encoding for β-tubulin
CBS: Acronym used for fungal strains preserved in Westerdijk Institute for Fungal Biodiversity Culture Collection, Utrecht, the Netherlands (former Centraalbureau voor Schimmelcultures)
D1/D2 of 28S: D1/D2domains of 28S rDNA
EXF: Acronym used for fungal strains preserved in the Microbial Culture Collection (Ex) of the Infrastructural Centre Mycosmo, MRIC UL, Slovenia
ITS: Internal transcribed spacer of rDNA
*MCM7*: Partial genes encoding for mini-chromosome maintenance protein
